# In Vitro and In Vivo Antiplasmodial Activities, Phytochemical Characterization, and Acute Oral Toxicity of Leaf Extracts of *Blumea balsamifera*

**DOI:** 10.3390/plants15203118

**Published:** 2026-10-12

**Authors:** Arisara Phuwajaroanpong, Chuchard Punsawad, Tanwarat Thanwisate, Prapaporn Chaniad, Atthaphon Konyanee, Piyakorn Boonyoung, Abdi Wira Septama, Walaiporn Plirat

**Affiliations:** 1 Department of Medical Technology, School of Allied Health Sciences, Walailak University, Nakhon Si Thammarat 80160, Thailand; arisara.pw@wu.ac.th; 2 Center of Excellence in Tropical Pathobiology, Walailak University, Nakhon Si Thammarat 80160, Thailand; chuchard.pu@wu.ac.th (C.P.); tanwarat.th@wu.ac.th (T.T.); prapaporn.ch@wu.ac.th (P.C.); atthaphon.ko@wu.ac.th (A.K.); 3 Department of Medical Sciences, School of Medicine, Walailak University, Nakhon Si Thammarat 80160, Thailand; 4 Division of Health and Applied Sciences, Faculty of Science, Prince of Songkla University, Songkhla 90110, Thailand; piyakorn.b@psu.ac.th; 5 Research Center for Pharmaceutical Ingredient and Traditional Medicine, Cibinong Science Center, National Research and Innovation Agency (BRIN), Cibinong Science Center, Cibinong, Bogor 16915, Indonesia; abdiwiraseptama@gmail.com

**Keywords:** *Blumea balsamifera*, antiplasmodial activity, toxicity, malaria

## Abstract

*Blumea balsamifera* is traditionally used in Thai herbal remedies for fever management; however, its antiplasmodial potential has not been comprehensively investigated. In this study, we evaluated the in vitro and in vivo antiplasmodial activities, phytochemical profile, and acute oral toxicity of *B. balsamifera* leaf extracts. Antiplasmodial activity against *Plasmodium falciparum* K1 and cytotoxicity against Vero cells were determined using the parasite lactate dehydrogenase (pLDH) assay and an MTT assay, respectively. Phytochemical constituents of the ethanolic extract were characterized by liquid chromatography–quadrupole time-of-flight mass spectrometry (LC-QTOF-MS). In vivo antiplasmodial assessment was investigated in *Plasmodium berghei* ANKA-infected mice using Peters’ four-day suppressive test at oral doses of 200, 400, and 600 mg/kg. Acute oral toxicity was evaluated following a single oral dose of 2000 mg/kg. The ethanolic extract exhibited promising in vitro antiplasmodial activity, with an IC_50_ value of 2.65 µg/mL and a selectivity index (SI) of 23.03, whereas the aqueous extract was inactive (IC_50_ > 40 µg/mL). LC-QTOF-MS analysis of the ethanolic leaf extract tentatively identified 16 compounds in the negative ionization mode and 30 compounds in the positive ionization mode. In the four-day suppressive test, the ethanolic extract demonstrated dose-dependent antiplasmodial activity, with parasitemia suppression of 42.29%, 70.74%, and 76.54% at doses of 200, 400, and 600 mg/kg, respectively. In the acute toxicity study, no mortality, abnormal clinical signs, or significant changes in body weight, food consumption, or water intake were observed. Furthermore, no evidence of hepatotoxicity or nephrotoxicity was detected. The ethanolic leaf extract of *B. balsamifera* demonstrated antiplasmodial activity in both in vitro and in vivo models, with favorable selectivity and no apparent acute toxicity at the tested dose. These findings support the potential of *B. balsamifera* as a source of antiplasmodial compounds and provide a basis for further phytochemical isolation and mechanistic studies.

## 1. Introduction

Malaria remains one of the most prominent infectious diseases worldwide and is caused by protozoan parasites of the genus *Plasmodium*. The disease is transmitted to humans through the bites of infected female *Anopheles* mosquitoes. Of the five *Plasmodium* species that infect humans—*P. falciparum*, *P. vivax*, *P. ovale*, *P. malariae,* and *P. knowlesi*—*P. falciparum* is the most virulent and is responsible for the majority of malaria-related deaths worldwide [1]. Severe *P. falciparum* infection may progress to severe anemia, metabolic acidosis, respiratory distress, cerebral malaria, and multiple-organ dysfunction [2]. Recent estimates from the World Health Organization indicate that malaria remains a major global public health concern, with approximately 282 million cases and 610,000 deaths in 2024. The disease burden is disproportionately concentrated in low- and middle-income regions, particularly in sub-Saharan Africa, Southeast Asia, and some areas of South America [3]. Artemisinin-based combination therapies (ACTs) are currently recommended as the first-line treatment for uncomplicated *P. falciparum* malaria, while parenteral artesunate followed by a complete oral ACT regimen is the preferred treatment for severe malaria [4]. Other antimalarial drugs, including chloroquine, primaquine, and tafenoquine, are used in selected clinical settings, depending on the infecting species, local drug-susceptibility patterns, glucose-6-phosphate dehydrogenase status, and national treatment guidelines [5,6]. Despite the effectiveness of these therapeutic regimens, the increasing emergence and spread of resistance to artemisinin derivatives and their partner drugs pose a major threat to malaria treatment and control.

The emergence and dissemination of drug-resistant *P. falciparum* parasites have posed a major challenge to global malaria control and elimination efforts [7]. Chloroquine, once the cornerstone of antimalarial therapy, has largely lost its efficacy against *P. falciparum* due to widespread resistance, although it continues to be effective against *P. vivax* and *P. malariae* in certain endemic regions [8]. In response, ACTs were developed by combining rapidly acting artemisinin derivatives (e.g., artesunate and dihydroartemisinin) with longer-acting partner drugs such as piperaquine, lumefantrine, and mefloquine to enhance therapeutic efficacy and delay resistance development [9]. Despite these advances, reduced susceptibility to artemisinin and declining efficacy of partner drugs are increasingly being reported, particularly in the Greater Mekong Subregion, including Thailand, Cambodia, and Myanmar [10]. Artemisinin-resistant parasites have also been detected in multiple countries with low transmission intensity, including Rwanda, Uganda, Ethiopia, Sudan, Kenya, and the eastern Democratic Republic of Congo, suggesting a concerning geographic expansion of resistance [11]. Taken together, these findings highlight the growing threat of treatment failure and underscore the urgent need for novel antimalarial strategies.

Considering these challenges, there is an urgent need to identify and develop novel antimalarial agents capable of overcoming therapeutic limitations and improving disease control. Among the most promising avenues for drug discovery are traditional medicinal plants, which represent a rich source of bioactive compounds with diverse pharmacological properties [12]. These natural resources have historically contributed to major antiplasmodial breakthroughs and continue to offer significant potential for the development of new, effective therapies [13]. *Blumea balsamifera* (L.) DC. (*B. balsamifera*), a member of the family *Asteraceae*, has long been recognized as an important medicinal plant in traditional healthcare systems. This species has been utilized for several centuries, reflecting its enduring ethnopharmacological relevance. *B. balsamifera* is widely distributed and traditionally employed across multiple countries in Southeast Asia, including China, Malaysia, Vietnam, the Philippines, and Thailand [14,15]. In these regions, it has been incorporated into various traditional remedies, highlighting its broad cultural acceptance and potential therapeutic significance. The extensive historical use of this plant underscores its value as a promising source for further pharmacological investigation and drug development [16]. Ethnomedical evidence indicates that *B. balsamifera* has been traditionally used to treat conditions such as sinusitis, colic pain, cough, kidney stones, and influenza-like illnesses, as well as to promote diuresis. Phytochemical studies have revealed that this plant contains a diverse range of volatile compounds (e.g., terpenoids, fatty acids, phenols, and aldehydes) and non-volatile constituents, particularly flavonoids, flavanones, and chalcones [17,18]. Consistent with its chemical diversity, extracts and bioactive compounds from *B. balsamifera* exhibit multiple pharmacological activities, including antioxidant, antimicrobial, antifungal, and anti-inflammatory effects, as well as hypolipidemic, hepatoprotective, antidiabetic, and gastroprotective properties. Additionally, its potential antitumor, anticancer, and immunomodulatory activities—particularly in the context of COVID-19—have been increasingly reported [17]. Despite its extensive traditional use and documented pharmacological activities, relatively few studies have investigated the antiplasmodial potential of *B. balsamifera*. Given its ethnomedicinal relevance and diverse bioactive properties, this plant represents a promising candidate for the development of alternative or complementary therapeutic strategies for the management of malaria.

In this study, we systematically investigated the antiplasmodial potential of *B. balsamifera* leaf extracts by evaluating ethanolic and aqueous extracts for their in vitro activity against *P. falciparum*. Building upon these findings, the most promising extract was further examined for its in vivo antiplasmodial efficacy using a *P. berghei*-infected murine model. In addition, an acute toxicity assessment was conducted in mice to determine the preliminary safety profile of the selected extract.

## 2. Materials and Methods

### 2.1. Plant Materials

The dried leaves of *B. balsamifera* were obtained from a traditional Thai pharmacy located in Mueang District, Nakhon Si Thammarat Province, Southern Thailand. The *B. balsamifera* (SMD072012003) specimens were taxonomically authenticated and scientifically identified by a specialist botanist affiliated with the School of Pharmacy, Walailak University, to ensure accurate botanical identification. Voucher specimens were subsequently deposited in the Department of Medical Sciences, School of Medicine, Walailak University, Thailand.

### 2.2. Plant Material Preparation, Extraction, and Percentage Yield Determination

The extraction procedure was performed according to a previously reported method [19]. Initially, the plant materials were washed with water to remove impurities and then dried in a hot-air oven at 60 °C for 72 h. After drying, the samples were chopped using a grinder (Taizhou Jincheng Pharmaceutical Machinery Co., Ltd., Model SF, Taizhou, China). Subsequently, 60 g of each dried sample was extracted using two different techniques: maceration and decoction. For the maceration method, 60 g of dried plant material was soaked in 600 mL of 95% ethanol (Merck, Darmstadt, Germany) at room temperature for 72 h. For the decoction method, 60 g of the dried sample was boiled in 600 mL of distilled water at 90–100 °C for 30 min. Each extraction process was performed twice to maximize extraction efficiency. The resulting extracts were filtered through Whatman No. 1 filter paper (GE Healthcare, Chicago, IL, USA). The ethanolic extract was concentrated using a rotary evaporator (Heidolph Hei-VAP, Schwabach, Germany) at 45 °C. Both the ethanolic and aqueous extracts were subsequently freeze-dried using a Christ Gamma 2–16 LSC plus freeze dryer (Christ, Osterode am Harz, Germany). Finally, the dried extracts were stored in airtight containers at 4–8 °C until further analysis. The percentage yield (%) was calculated as the amount of crude extract obtained relative to the initial weight of the plant material used for extraction [20], according to the following equation:

$$\%\;\mathrm{Yield}=\frac{\mathrm{Weight}\;\mathrm{of}\;\mathrm{dried}\;\mathrm{plant}\;\mathrm{extract}\left(g\right)}{\mathrm{Initial}\;\mathrm{weight}\;\mathrm{of}\;\mathrm{plant}\;\mathrm{material}\left(g\right)}\times 100$$

### 2.3. Ethical Considerations

The study protocol involving human subjects was reviewed and approved by the Walailak University Human Research Ethics Committee (approval No. WUEC-26-127-01). Written informed consent was obtained from all participants prior to blood collection for malaria parasite cultivation, antiplasmodial activity testing, and hemolysis assay. All laboratory procedures involving malaria parasites and human blood samples were conducted in accordance with institutional biosafety regulations under Biosafety Level 2 (BSL-2) containment conditions and were approved by the Walailak University Biosafety Committee (approval No. WU-IBC-69-001). Animal experiments were approved by the Walailak University Institutional Animal Care and Use Committee (approval No. WU-ACUC-69003). All procedures were conducted in accordance with institutional guidelines and the ARRIVE guidelines. Animals were closely monitored throughout the study, and every effort was made to minimize pain, distress, and suffering, including the use of isoflurane anesthesia when appropriate.

### 2.4. In Vitro Culture and Propagation of the Malaria Parasite

The chloroquine-resistant *P. falciparum* K1 strain was kindly provided by Dr. Rapatbhorn Patrapuvich of Mahidol University, Thailand, and maintained according to the method originally described by Trager and Jensen [21]. Parasites were cultivated in Roswell Park Memorial Institute (RPMI) 1640 medium (Gibco, New York, NY, USA) supplemented with 2 mg/mL sodium bicarbonate (Sigma-Aldrich, St. Louis, MO, USA) to regulate pH, 10 µg/mL, hypoxanthine (Sigma-Aldrich, St. Louis, MO, USA) to facilitate purine metabolism, and 4.8 mg/mL HEPES buffer (Himedia, Mumbai, India) to stabilize the pH under atmospheric conditions. In addition, 0.5% (*w*/*v*) AlbuMAX™ II, a lipid-rich bovine serum albumin (Gibco, Auckland, New Zealand), was added as a source of essential proteins and lipids for parasite growth. To minimize bacterial contamination, the medium was supplemented with 2.5 µg/mL gentamicin (Sigma-Aldrich, Mumbai, India). Parasitemia was monitored by preparing Giemsa-stained thin blood smears and examining them under a light microscope (Olympus CX31, Tokyo, Japan) using a 100× oil- immersion objective lens. Parasite cultures were routinely subcultured every 24–48 h by adjusting the hematocrit level and renewing the culture medium. For stage synchronization, ring-stage parasites were obtained using a 5% D-sorbitol treatment, which selectively lyses mature trophozoite and schizont stages while preserving ring-stage parasites. The synchronized parasites were then washed, resuspended in complete culture medium, and maintained for subsequent experiments.

### 2.5. In Vitro Evaluation of Antiplasmodial Activity

The antiplasmodial activity of ethanolic and aqueous extracts of *B. balsamifera* leaves was evaluated using the pLDH assay originally developed by Michael T. Makler [22]. This method uses the amount of pLDH enzyme produced by viable *P. falciparum* parasites as an indicator of parasite viability. Stock solutions of test samples were prepared by dissolving the extracts in dimethyl sulfoxide (DMSO; Merck, Darmstadt, Germany) and RPMI 1640 medium. The solutions were then serially diluted to obtain final concentrations ranging from 1.56 to 100 µg/mL. Artesunate (Sigma-Aldrich, St. Louis, MO, USA) was used as the positive control, whereas DMSO served as the negative control and uninfected erythrocytes were used as the baseline reference. Cultures of *P. falciparum* K1-infected erythrocytes were adjusted to 2% parasitemia and 2% hematocrit, and 199 µL of the culture suspension was dispensed into each well of a 96-well microplate. Test extracts, controls, and vehicle solutions (1 µL/well) were added in triplicate. The plates were incubated at 37 °C in a humidified atmosphere containing 5% CO_2_ for 72 h. Following incubation, parasite-infected erythrocytes were subjected to three freeze–thaw cycles (−20 °C for 30 min followed by 37 °C for 30 min) to lyse the cells and release pLDH. Subsequently, 20 µL of the lysate was transferred to a new 96-well microplate, and 100 µL of Malstat reagent was added to catalyze the conversion of lactate to pyruvate by pLDH. Thereafter, 20 µL of chromogenic detection solution containing p-nitroblue tetrazolium chloride (NBT; Merck, Darmstadt, Germany) and phenazine ethosulfate (PES; Sigma-Aldrich, St. Louis, MO, USA) was added to each well to enable colorimetric detection through the formation of a purple formazan product. The plates were incubated in the dark at room temperature for 1 h. Absorbance was measured at 650 nm using a Multiskan SkyHigh microplate spectrophotometer (Thermo Fisher Scientific, Waltham, MA, USA). The half-maximal inhibitory concentration (IC_50_) values of the extracts were calculated using nonlinear regression analysis with GraphPad Prism version 6 (GraphPad Software, Boston, MA, USA). Antiplasmodial activity was classified according to previously established criteria [23] as highly active (IC_50_ < 5 µg/mL), active (5–10 µg/mL), moderately active (10–20 µg/mL), weakly active (20–40 µg/mL), or inactive (IC_50_ > 40 µg/mL).

### 2.6. Culture and Maintenance of Vero Cells

Vero cells (ATCC^®^ CCL-81™, RRID: CVCL_0059), originally derived from kidney epithelial cells of an adult African green monkey (*Chlorocebus sabaeus*), were cultured in Dulbecco’s Modified Eagle’s Medium (DMEM; Gibco, New York, NY, USA) supplemented with 10% heat-inactivated fetal bovine serum (FBS; Sigma-Aldrich, Mumbai, India) and 1% penicillin–streptomycin. Cells were maintained at 37 °C in a humidified incubator with 5% CO_2_. Cell morphology and confluence were routinely examined using a phase-contrast inverted microscope (Olympus CKX31, North Brooklyn Park, MN, USA). When cultures reached approximately 80% confluence, cells were detached using 2.5% trypsin–EDTA (Gibco, New York, NY, USA), neutralized with complete medium, and passaged at an appropriate split ratio to maintain continuous growth. All procedures were performed under aseptic conditions.

### 2.7. In Vitro Evaluation of Cytotoxicity

The cytotoxicity of ethanolic and aqueous extracts of *B. balsamifera* leaves was evaluated in vitro using the 3-(4,5 dimethylthiazol-2-yl)-2,5-diphenyltetrazolium bromide (MTT) assay following a previously described protocol with minor modifications [24]. For the assay, Vero cells were seeded into 96-well plates at a density of 1 × 10^4^ cells per well in 199 μL of complete medium and allowed to adhere overnight. Subsequently, 1 μL of each test substance dissolved in DMSO and DMEM were added to obtain final concentrations ranging from 1.56 to 100 μg/mL. All treatments were performed in triplicate. Doxorubicin (DX; Sigma-Aldrich, Mumbai, India) was used as a positive control, whereas DMSO and DMEM served as the negative control. Following 48 h of incubation, the culture medium was carefully removed, and 50 μL of MTT solution (5 mg/mL in PBS; Thermo Fisher Scientific, Waltham, MA, USA) was added to each well. The plates were incubated for an additional 2 h to allow the formation of formazan crystals by metabolically active cells. The supernatant was then discarded, and the resulting formazan crystals were dissolved in 100 μL of DMSO. Absorbance was measured at 560 nm using a Multiskan SkyHigh spectrophotometer (Thermo Fisher Scientific, Waltham, MA, USA), and background absorbance at 670 nm was subtracted. The 50% cytotoxic concentration (CC_50_) values were calculated by nonlinear regression using GraphPad Prism (GraphPad Software, Boston, MA, USA).

### 2.8. Determination of the Selectivity Index (SI)

The selectivity index (SI) was calculated to identify potentially selective crude extracts and compounds from *B. balsamifera* for further in vivo antiplasmodial evaluation. An SI value greater than 10 is commonly regarded as indicative of a favorable therapeutic window, representing a substantial margin between antimalarial activity and cytotoxicity toward mammalian cells [25]. Therefore, crude extracts exhibiting SI values above this threshold together with strong in vitro antiplasmodial activity were prioritized for subsequent in vivo efficacy and safety studies. The SI was calculated as the ratio of the CC_50_ in Vero cells to the IC_50_ against *P. falciparum*, as shown in the following equation [26]:

$$\mathrm{Selectivity}\;\mathrm{index}\;(\mathrm{SI})\;=\frac{{\mathrm{CC}}_{50}\;(\mathrm{Vero}\;\mathrm{cells})}{{\mathrm{IC}}_{50}\;(P.\;\mathrm{falciparum}\;K1)}$$

### 2.9. Human Erythrocyte Hemolysis Assay

The cytotoxicity of ethanolic and aqueous extracts of *B. balsamifera* leaves toward human erythrocytes was evaluated using a hemolysis assay. After blood collection from the participants, red blood cells (RBCs) were isolated by centrifugation at 3000 rpm for 5 min, followed by removal of plasma and buffy coat layers. The RBC pellet was washed three times with sterile phosphate-buffered saline (PBS) to remove plasma proteins and residual leukocytes. A 2% hematocrit RBC suspension was then prepared. For the hemolysis assay, the RBC suspension was incubated with 50 µg/mL of each plant crude extract in a final volume of 0.2 mL in sterile microplates. Samples were incubated at 37 °C for 24, 48, and 72 h under humidified conditions. Triton X-100 (Sigma-Aldrich, New Delhi, India) served as the positive control to induce complete hemolysis, while DMSO and PBS were used as negative controls. After incubation, plates were centrifuged at 3000 rpm for 5 min to pellet intact RBCs. The supernatant was transferred to a new 96-well plate, and hemoglobin release was quantified by measuring absorbance at 570 nm using a microplate spectrophotometer. Extracts inducing <10% hemolysis were considered non-hemolytic, whereas those causing >25% hemolysis were regarded as having significant hemolytic activity against healthy human RBCs. The percentage of hemolysis was calculated using the following formula [27]:

$$\%\;\mathrm{Hemolysis}=\frac{\left(\mathrm{Optical}\;\mathrm{density}\;\mathrm{of}\;\mathrm{sample}-\mathrm{Optical}\;\mathrm{density}\;\mathrm{of}\;\mathrm{negative}\;\mathrm{control}\right)}{\left(\mathrm{Optical}\;\mathrm{density}\;\mathrm{of}\;\mathrm{positive}\;\mathrm{control}-\mathrm{Optical}\;\mathrm{density}\;\mathrm{of}\;\mathrm{negative}\;\mathrm{control}\right)}\times 100$$

### 2.10. Phytochemical Identification on B. balsamifera Leaves

Phytochemical profiling of the selected plant extract was performed using liquid chromatography–quadrupole time-of-flight mass spectrometry (LC-QTOF-MS) on a 1290 Infinity II LC system coupled to a 6545 Quadrupole-TOF mass spectrometer (Agilent Technologies, Santa Clara, CA, USA) equipped with an electrospray ionization (ESI) source. Chromatographic separation was achieved using a Zorbax Eclipse Plus C18 column (100 × 2.1 mm, 1.8 μm; Agilent Technologies, Santa Clara, CA, USA) maintained at 25 °C. The mobile phase consisted of (A) 2% acetic acid in water and (B) acetonitrile, delivered at a flow rate of 0.20 mL/min with an injection volume of 2 μL. Mass spectral data were acquired in negative and positive ionization modes over an *m*/*z* range of 100–1200. For MS/MS analysis, the instrument automatically selected the most intense precursor ions within the specified mass range for fragmentation to facilitate structural characterization. Putative compound identification was achieved by comparing retention times and mass spectral data with reference spectra available in the METLIN database (Agilent Technologies, Santa Clara, CA, USA). Only compounds with similarity scores exceeding 80% were considered for annotation. Compounds with identical or closely related precursor masses were treated as distinct chromatographic features when they exhibited different retention times and/or characteristic MS/MS fragmentation patterns and were putatively differentiated using these data in conjunction with library matching. Retention time was used to distinguish chromatographic features rather than as definitive evidence of compound identity. Because authentic reference standards and stereochemical analyses were not employed, all compound assignments were considered putative annotations rather than definitive structural identifications, and specific stereochemical configurations were not assigned.

### 2.11. Animal Acclimatization Prior to the 4-Day Suppressive Test

Male Institute of Cancer Research (ICR) mice (also known as CD-1 mice; RRID: IMSR_CRL:022), an albino strain of *Mus musculus* derived from the Swiss lineage, were used in this study. Animals aged 6–8 weeks and weighing 20–30 g were obtained from Nomura Siam International Co., Ltd. (Bangkok, Thailand). Mice were housed under controlled conditions with a 12 h light/dark cycle, a temperature of 22 ± 3 °C, and a relative humidity of 50–60%. Animals received a standard commercial pellet diet and water ad libitum. Prior to experimentation, mice were acclimatized for one week to minimize stress. Animals were randomly assigned to experimental groups, and all procedures were conducted in accordance with international guidelines for the ethical use and care of laboratory animals.

### 2.12. In Vivo Antiplasmodial Assessment Using the Peters’ 4-Day Suppressive Test

In vivo antiplasmodial activity was evaluated using the Peters’ 4-day suppressive test. The rodent malaria parasite *P. berghei* ANKA was obtained from BEI Resources, National Institute of Allergy and Infectious Diseases, National Institutes of Health (originally provided by Thomas F. McCutchan). Parasites were maintained in donor ICR mice by intraperitoneal inoculation of infected erythrocytes from frozen stocks. Parasitemia was monitored using Giemsa-stained thin blood smears, and donor blood was collected when parasitemia reached 20–30%. Donor mice were anesthetized with 2% isoflurane (Piramal Pharma, Sellersville, PA, USA) until loss of reflexes was confirmed, followed by cardiac puncture and euthanasia according to institutional animal care regulations. Collected blood was diluted with 0.9% normal saline to prepare an inoculum containing 1 × 10^7^ parasitized erythrocytes in 0.2 mL. Thirty mice were randomly assigned to six groups (n = 5 per group). The uninfected control group received 7% Tween 80 orally. The remaining twenty-five mice were intraperitoneally inoculated with 0.2 mL of the prepared parasite suspension and allocated to five infected groups. The negative control group received 7% Tween 80, while the positive control group was treated orally with chloroquine (25 mg/kg body weight). The treatment groups received the selected crude extract orally at doses of 200, 400, or 600 mg/kg body weight. Treatments were administered at 3, 24, 48, and 72 h post-infection. On day 4, all mice were anesthetized with 2% isoflurane (Piramal Pharma, Sellersville, PA, USA) and euthanized by cardiac puncture. Thin blood smears were prepared, fixed with methanol, stained with Giemsa, and examined under a light microscope (Olympus CX31, Olympus Corp., Tokyo, Japan) using a 100× oil immersion objective. Parasitemia was determined by counting parasitized erythrocytes among the total number of erythrocytes examined. The percentage of parasitemia was calculated using the following equation [28]:

$$\%\;\mathrm{Parasitemia}=\frac{\mathrm{Number}\;\mathrm{of}\;\mathrm{parasitized}\;\mathrm{RBCs}}{\mathrm{Total}\;\mathrm{number}\;\mathrm{of}\;\mathrm{RBCs}\;\mathrm{counted}}\times 100$$

The percentage suppression of parasitemia was calculated relative to the negative control group using the following equation:

$$\%\;\mathrm{Parasitemia}\;\mathrm{suppression}=\frac{\mathrm{Mean}\;\mathrm{parasitemia}\;\mathrm{of}\;\mathrm{negative}\;\mathrm{control}-\mathrm{Mean}\;\mathrm{parasitemia}\;\mathrm{of}\;\mathrm{treatment}\;\mathrm{group}}{\mathrm{Mean}\;\mathrm{parasitemia}\;\mathrm{of}\;\mathrm{negative}\;\mathrm{control}}\times 100$$

Following blood collection, samples were analyzed for hematological and biochemical parameters. RBC count, hemoglobin (Hb) concentration, hematocrit (HCT), platelet count, and white blood cell (WBC) count were determined using an automated hematology analyzer. Serum alanine aminotransferase (ALT), aspartate aminotransferase (AST), and alkaline phosphatase (ALP) levels were measured using an AU480 automated chemistry analyzer (Beckman Coulter, Brea, CA, USA). The liver and spleen were subsequently excised, fixed in 10% paraformaldehyde, and processed for histopathological examination.

### 2.13. Measurement of Body Weight Changes

Body weight was recorded using a calibrated digital analytical balance on Day 0 (before infection) and Day 4 (after treatment). The percentage change in body weight for each mouse was calculated using the following equation [29]:

$$\%\;\mathrm{Body}\;\mathrm{weight}\;\mathrm{change}=\frac{\mathrm{Body}\;\mathrm{weight}\;\mathrm{on}\;\mathrm{day}\;4-\mathrm{Body}\;\mathrm{weight}\;\mathrm{on}\;\mathrm{day}\;0}{\mathrm{Body}\;\mathrm{weight}\;\mathrm{on}\;\mathrm{day}\;0}\times 100$$

### 2.14. Evaluation of Acute Oral Toxicity in ICR Mice

Acute oral toxicity was evaluated in accordance with OECD Guideline 425 to assess the safety profile of the selected crude extract [30]. Fifteen male ICR mice were randomly assigned to three groups (n = 5 per group): a normal control group, a vehicle control group receiving 7% Tween 80, and a treatment group administered a single oral dose of the crude extract at 2000 mg/kg body weight. The crude extract was freshly prepared by suspending the dried extract in 7% Tween 80 to obtain the desired concentration. Mice in the treatment group received the extract by oral gavage, whereas the vehicle control group received an equivalent volume of 7% Tween 80.

Animals were fasted for 2 h before dosing and observed continuously during the first 24 h and daily thereafter for 14 days for clinical signs of toxicity, including behavioral, neurological, gastrointestinal, and physiological abnormalities. Food and water intake were recorded daily, and body weight was measured on days 0 (pre-treatment), 7, and 14 using a calibrated digital analytical balance.

On day 14, all animals were anesthetized with 2% isoflurane (Piramal Pharma, Sellersville, PA, USA) using a rodent anesthesia system and euthanized after adequate anesthesia was confirmed by the absence of toe-pinch and corneal reflexes. Blood samples were collected for biochemical analysis of hepatic and renal function. The liver and kidneys were excised, rinsed with normal saline, and fixed in 10% paraformaldehyde for subsequent histopathological examination. Histopathological evaluation was independently performed by two blinded histopathologists using light microscopy. Hepatic and renal sections were assessed for pathological changes, including cellular degeneration, necrosis, inflammatory cell infiltration, and disruption of normal tissue architecture. The findings were used to evaluate potential tissue toxicity associated with administration of the crude extract.

### 2.15. Biochemical Analysis of Acute Oral Toxicity

On day 14 of the acute oral toxicity study, blood samples were collected following euthanasia for biochemical evaluation of hepatic and renal function. Blood was transferred into clot activator tubes and centrifuged at 3000 rpm for 5 min to obtain serum. Serum biochemical parameters were analyzed using an AU480 automated chemistry analyzer (Beckman Coulter, Brea, CA, USA). Hepatic function was evaluated by measuring serum ALT, AST, and ALP activities. Renal function was assessed by determining blood urea nitrogen (BUN) and serum creatinine concentrations. All analyses were performed in accordance with the manufacturer’s instructions and standardized operating procedures to ensure the accuracy, precision, and reproducibility of the results.

### 2.16. Data Analysis and Statistical Methods

G* Power program version 3.1.9.7 was used to calculate the sample size for animal in 4-day suppressive and acute toxicity study. The effect size was set at f = 0.80, with a test power of 0.95 and a significance level (α) of 0.05. All statistical analyses were performed using SPSS version 29.0 (IBM Corp., Armonk, NY, USA). Data are presented as the mean ± standard deviation (SD). Normality of the data was assessed using the Kolmogorov–Smirnov test before statistical analysis. The half-maximal inhibitory concentration (IC_50_) and half-maximal cytotoxic concentration (CC_50_) were determined by nonlinear regression analysis using GraphPad Prism version 6.0 (GraphPad Software, San Diego, CA, USA). Differences among experimental groups, including parasitemia, percentage suppression of parasitemia, food and water consumption, body weight, hematological parameters, and biochemical indices, were analyzed using one-way analysis of variance (ANOVA), followed by Tukey’s post hoc multiple comparison test when appropriate. A *p*-value < 0.05 was considered statistically significant.

## 3. Results

### 3.1. Percentage Yield of B. balsamifera Leaf Extracts

The aqueous leaf extract exhibited a higher extraction yield (22.72%) than the ethanolic leaf extract (16.33%), with yields calculated based on the dry weight of the starting plant material. Both crude extracts demonstrated good solubility in their respective solvents and were considered suitable for subsequent in vitro and in vivo assessments of antiplasmodial activity and toxicity.

### 3.2. Antiplasmodial Activity, Cytotoxicity, and Selectivity Index of B. balsamifera Leaf Extracts

The in vitro antiplasmodial activity of *B. balsamifera* leaf extracts against *P. falciparum* is presented in Table 1. The ethanolic extract exhibited strong antiplasmodial activity, with an IC_50_ value of 2.65 µg/mL and was therefore classified as highly active. In contrast, the aqueous extract demonstrated much lower activity, with an IC_50_ value of 84.54 µg/mL, corresponding to inactive antiplasmodial activity according to the stated classification criteria.

Cytotoxicity against Vero cells was evaluated according to the U.S. National Cancer Institute (NCI) criteria, which classify crude extracts with CC_50_ values < 30 µg/mL after 48–72 h of exposure as cytotoxic. Both extracts exhibited CC_50_ values above this threshold, indicating low cytotoxicity. The ethanolic extract showed a CC_50_ value of 61.03 µg/mL, whereas the aqueous extract also demonstrated low cytotoxicity.

The SI, calculated as the ratio of CC_50_ to IC_50_, was used to assess the selectivity of the extracts toward *P. falciparum* relative to Vero cells. The ethanolic extract exhibited an SI value of 23.03, indicating favorable selectivity and a substantially greater inhibitory effect on the parasite than on mammalian cells. In contrast, the aqueous extract showed a markedly lower SI value (>2.36) because of its limited antiplasmodial activity. Taken together, these findings demonstrate that extraction solvent markedly influenced the biological activity of *B. balsamifera*, with the ethanolic extract displaying greater antiplasmodial activity while maintaining low cytotoxicity. Given its favorable selectivity and strong in vitro antiplasmodial activity, the ethanolic extract was selected for subsequent in vivo assessments and safety evaluation.

### 3.3. Hemolytic Activity of B. balsamifera Leaf Extracts

**Figure 1:**
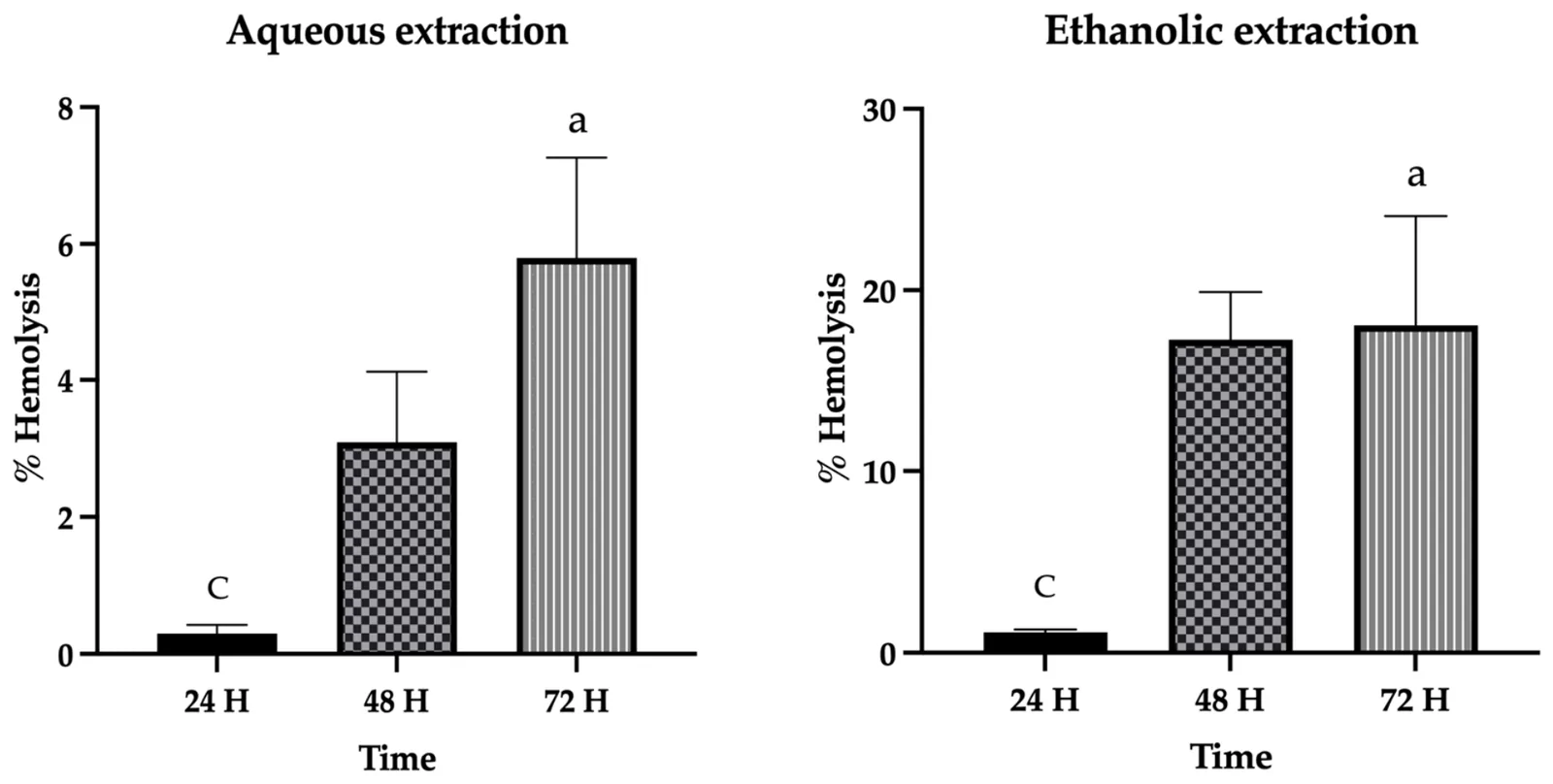
Hemolytic activity of *B. balsamifera* leaf extracts in an in vitro hemolysis assay. Data are presented as the mean ± standard deviation (SD). Statistical significance was determined by one-way ANOVA followed by Tukey’s multiple comparisons test. a *p* < 0.05 compared with the 24 h incubation; c *p* < 0.05 compared with the 72 h incubation.

The hemolytic activity of *B. balsamifera* leaf extracts was evaluated at a concentration of 50 µg/mL (Figure 1). The aqueous extract did not induce appreciable hemolysis at any of the evaluated time points, indicating minimal effects on erythrocyte membrane integrity. In contrast, the ethanolic extract exhibited a time-dependent increase in hemolytic activity, with the percentage of hemolysis increasing from 1.12% after 24 h to 17.23% after 48 h and reaching 18.06% after 72 h of incubation. Based on the predefined criteria, the aqueous extract was considered non-hemolytic (<10%) at all time points. The ethanolic extract exhibited low hemolytic activity at 24 h but moderate hemolysis at 48 and 72 h, although the observed levels remained below the threshold for significant hemolytic activity (>25%). These findings indicate that both extracts had limited effects on erythrocyte membrane integrity under the experimental conditions, although the ethanolic extract showed a greater time-dependent hemolytic effect than the aqueous extract.

### 3.4. Chemical Characterization of the Ethanolic Leaf Extract of B. balsamifera in Negative Ionization Mode

**Figure 2:**
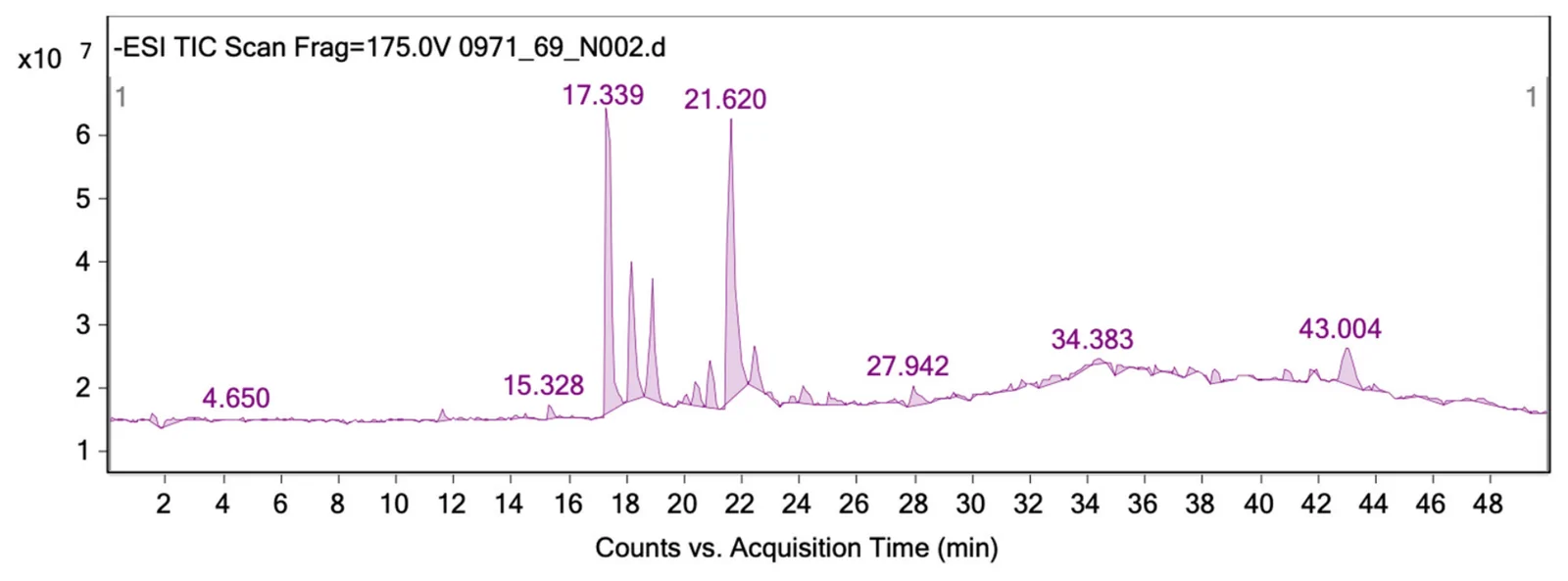
Full-scan LC-QTOF-MS chromatogram of the ethanolic leaf extract of *B. balsamifera* acquired in negative ionization mode.

Sixteen compounds were tentatively identified based on the LC-QTOF-MS analysis of the ethanolic leaf extract in negative ionization mode, predominantly comprising polar metabolites (Table 2). These included several flavonoids, namely taxifolin, luteolin, quercetin, apigenin, naringenin, diosmetin, hesperetin, prunetin, and koparin 2′-methyl ether. In addition, the acyl glycine derivative methyl N-(α-methylbutyryl)glycine was detected at a retention time (RT) of 16.146 min, and the polyphenolic metabolite lecanoric acid was identified at an RT of 19.028 min. The long-chain hydroxy fatty acid 2-hydroxyhexadecanoic acid was detected at a later retention time (RT = 34.311 min). Compounds 14 (13(S)-HOTrE) and 15 (9-OxoODE) share the same precursor ion and molecular formula. They were treated as distinct chromatographic features based on differences in retention times and MS/MS fragmentation patterns, with library matching used for putative annotation. Because authentic reference standards and stereochemical analyses were not employed, these annotations represent putative compound assignments and do not establish the definitive structural or stereochemical identities of the detected compounds. The corresponding LC-QTOF-MS chromatogram acquired in negative ionization mode is presented in Figure 2.

### 3.5. Chemical Characterization of the Ethanolic Leaf Extract of B. balsamifera in Positive Ionization Mode

**Figure 3:**
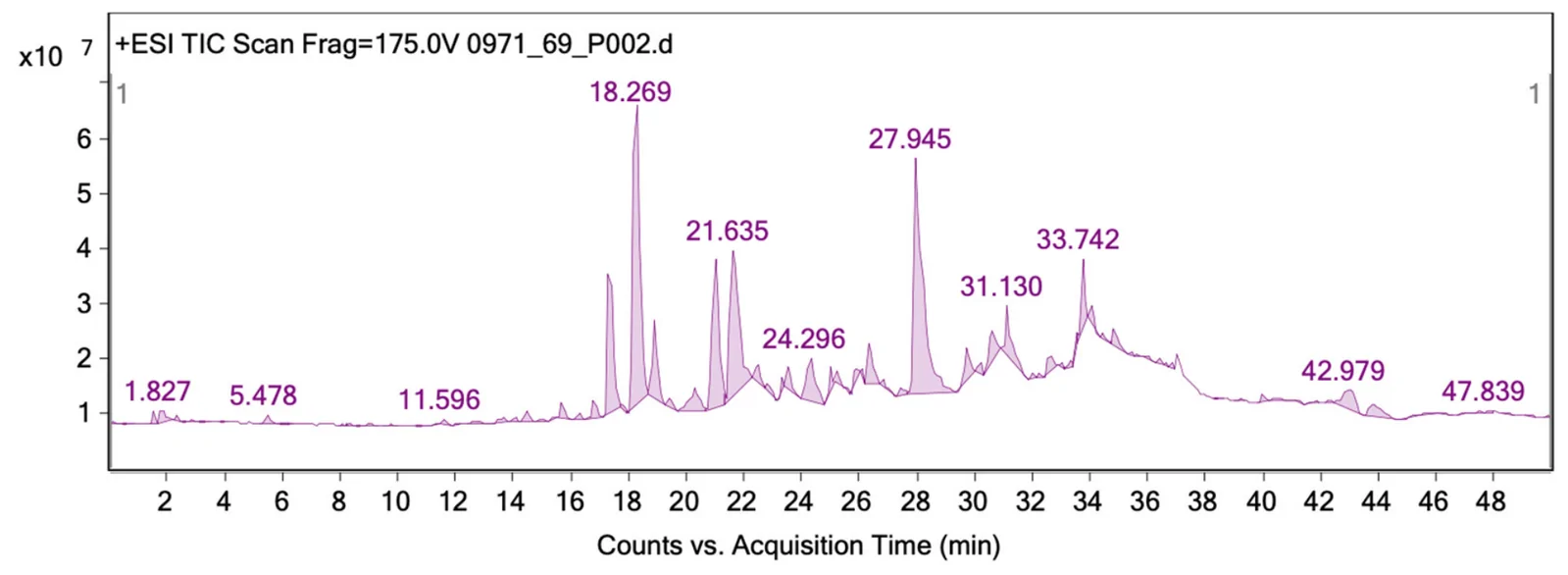
Full-scan LC-QTOF-MS chromatogram of the ethanolic leaf extract of *B. balsamifera* acquired in positive ionization mode.

LC-QTOF-MS analysis of the ethanolic leaf extract in positive ionization mode tentatively identified 30 compounds based on accurate mass measurements, isotopic distribution patterns, and database matching (Table 3). The detected constituents consisted predominantly of polar metabolites. Several flavonoids were tentatively identified, including taxifolin, naringenin, fisetin, quercetin 3-methyl ether, diosmetin, rhamnetin, glycitein, koparin 2′-methyl ether, and quercetin tetramethyl (5,7,3′,4′) ether. In addition, several terpenoid-related compounds were tentatively identified, including confertifoline, α-farnesene, and alantolactone, which eluted at RT of 18.543, 21.913, and 25.972 min, respectively. Putative alkaloids, including mitragynine (RT = 18.618 min) and quinine ethyl carbonate (RT = 20.523 min), were also detected. Furthermore, oleamide, a fatty acid amide derivative of oleic acid, was tentatively identified at a relatively late retention time (RT = 35.781 min), consistent with its lower polarity relative to the other detected metabolites. As no authentic reference standards or stereochemical analyses were employed, the LC-QTOF-MS results were considered putative annotations rather than definitive structural identifications. The corresponding LC-QTOF-MS chromatogram acquired in positive ionization mode is presented in Figure 3.

### 3.6. Four-Day Suppressive Antimalarial Activity

The in vivo antiplasmodial efficacy of the ethanolic leaf extract of *B. balsamifera* was evaluated in *P. berghei*-infected mice using the Peters’ four-day suppressive test (Table 4). The ethanolic extract was selected for in vivo evaluation based on its superior in vitro antiplasmodial activity compared with the aqueous extract. In the untreated infected (negative control) group exhibited a parasitemia level of 40.34 ± 5.16%, confirming successful establishment of infection. In contrast, chloroquine treatment (25 mg/kg) significantly reduced parasitemia to 0.80 ± 0.27%, corresponding to a parasite suppression rate of 98.01 ± 0.68% (*p* < 0.05 vs. negative control).

Treatment with the ethanolic extract resulted in a dose-dependent reduction in parasitemia. Mice treated with 200, 400, and 600 mg/kg exhibited parasitemia levels of 23.28 ± 0.94%, 11.80 ± 0.49%, and 9.46 ± 0.97%, respectively, corresponding to parasite suppression rates of 42.29 ± 2.33%, 70.74 ± 1.22%, and 76.54 ± 2.41%. All extract-treated groups showed significantly lower parasitemia than the negative control group (*p* < 0.05). Moreover, the 600 mg/kg dose produced significantly greater parasite suppression than the 200 mg/kg dose (*p* < 0.05), whereas no significant difference was observed between the 400 and 600 mg/kg treatment groups. These findings suggest that the antimalarial effect may have approached a plateau at higher doses.

Although the ethanolic extract significantly suppressed parasite growth, parasitemia remained detectable in all extract-treated groups, indicating that complete parasite clearance was not achieved during the four-day treatment period. Overall, the ethanolic extract demonstrated dose-dependent in vivo antimalarial activity, although its efficacy was lower than that of chloroquine.

### 3.7. Effect of B. balsamifera Leaf Extract on Hematological Parameters in the Four-Day Suppressive Test

**Figure 4:**
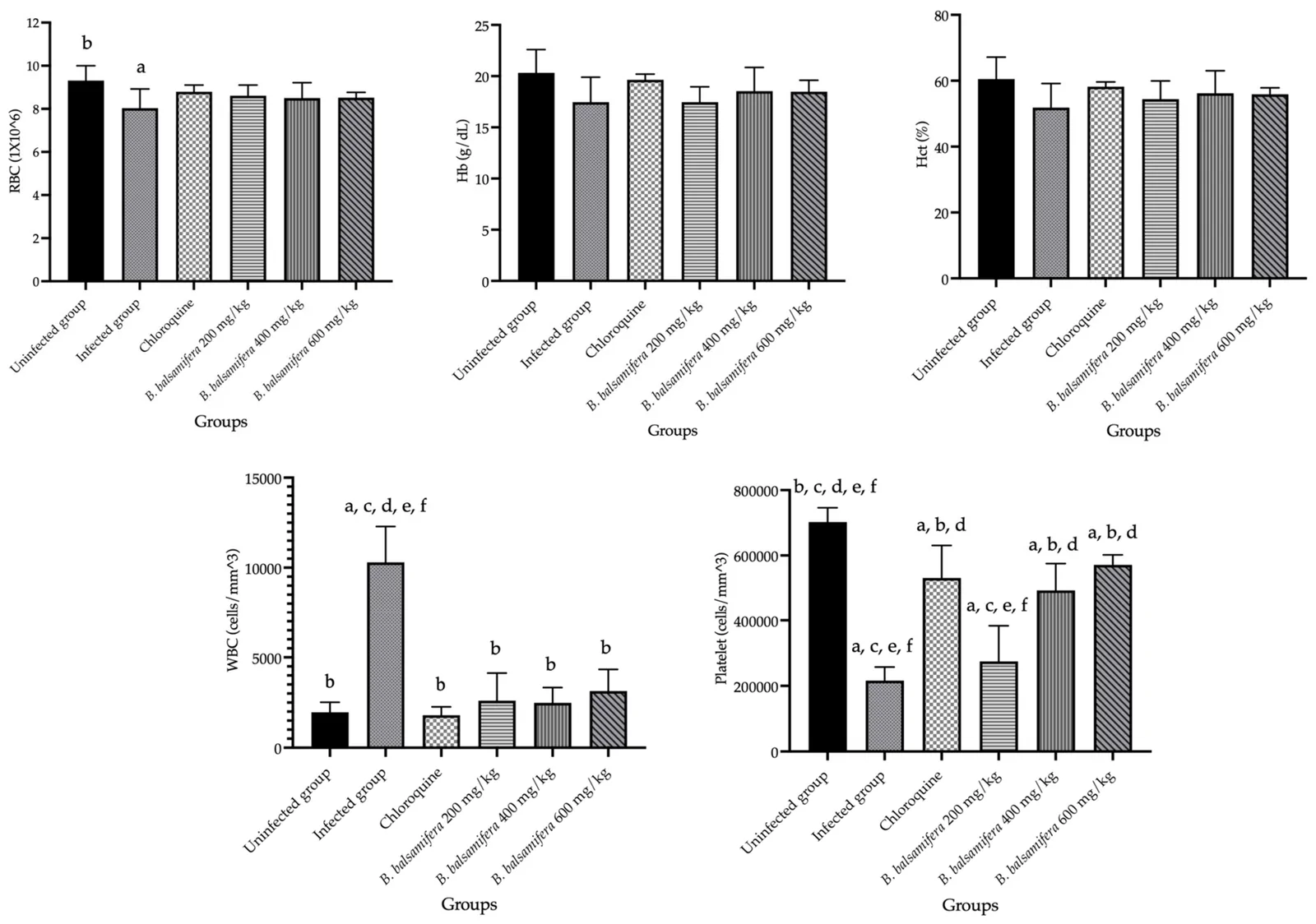
Effects of the ethanolic leaf extract of *B. balsamifera* on hematological parameters in the Peters’ four-day suppressive test. Red blood cell (RBC) count, hemoglobin (Hb) concentration, hematocrit (HCT), white blood cell (WBC) count, and platelet count were determined on Day 4. Data are presented as the mean ± standard deviation (SD) (n = 5 mice per group). Statistical differences among groups were analyzed using one-way analysis of variance (ANOVA) followed by Tukey’s multiple-comparison test. Lowercase letters above the bars indicate statistically significant differences (*p* < 0.05): a, compared with the uninfected control group; b, compared with the *P. berghei* ANKA-infected (negative control) group; c, compared with the chloroquine-treated group; d, compared with the 200 mg/kg *B. balsamifera* ethanolic leaf extract group; e, compared with the 400 mg/kg *B. balsamifera* ethanolic leaf extract group; and f, compared with the 600 mg/kg *B. balsamifera* ethanolic leaf extract group.

Hematological parameters following treatment are shown in Figure 4. The untreated *P. berghei* ANKA-infected group exhibited a significantly lower RBC count than the uninfected control group (*p* < 0.05). However, no statistically significant differences in Hb concentration or HCT were detected among the experimental groups (*p* > 0.05). In contrast, marked differences were observed in WBC counts. The untreated infected group exhibited a significantly higher WBC count than the uninfected control, chloroquine-treated group, and all *B. balsamifera* extract-treated groups (*p* < 0.05). WBC counts did not differ significantly between the uninfected control, chloroquine-treated, and extract-treated groups. Platelet counts were significantly reduced in the untreated infected group compared with the uninfected control (*p* < 0.05). Treatment with the extract at 400 and 600 mg/kg was associated with significantly higher platelet counts than those observed in the untreated infected group, whereas the 200 mg/kg group did not show a comparable increase. Nevertheless, platelet counts in the extract-treated groups remained significantly lower than those in the uninfected control group (*p* < 0.05). No significant difference was observed between the higher-dose extract groups and the chloroquine-treated group.

### 3.8. Effect of B. balsamifera Leaf Extract on Relative Liver and Spleen Weights in the Four-Day Suppressive Test

Table 5 shows the relative liver and spleen weights of *P. berghei* ANKA-infected mice following treatment with the ethanolic leaf extract of *B. balsamifera*. Malaria infection was associated with increased relative liver and spleen weights, with splenic enlargement being particularly pronounced. The untreated infected (negative control) group exhibited an approximately fourfold increase in relative spleen weight compared with the uninfected control group (*p* < 0.05). Treatment with the ethanolic leaf extract of *B. balsamifera* was associated with lower relative spleen weights compared with the untreated infected group. In particular, the 400 and 600 mg/kg extract-treated groups showed numerically lower relative spleen weights; however, these differences were not statistically significant. Relative liver weight also varied among the experimental groups, but no statistically significant differences were detected. The chloroquine-treated group (25 mg/kg) also showed lower relative spleen weights than the untreated infected group (*p* < 0.05).

### 3.9. Effect of B. balsamifera Leaf Extract on Liver Function Parameters in the Four-Day Suppressive Test

**Figure 5:**
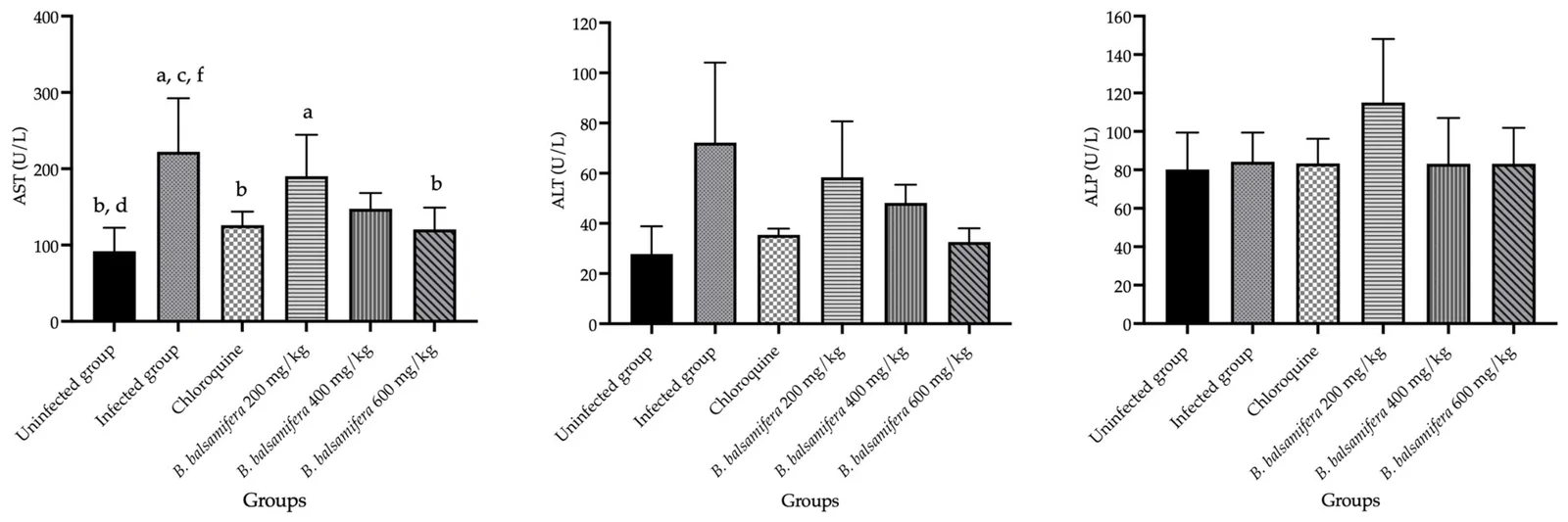
Effects of the ethanolic leaf extract of *B. balsamifera* on liver function parameters in the Peters’ four-day suppressive test. Serum aspartate aminotransferase (AST), alanine aminotransferase (ALT), and alkaline phosphatase (ALP) activities were determined on Day 4. Data are presented as the mean ± standard deviation (SD) (n = 5 mice per group). Statistical differences among groups were analyzed using one-way analysis of variance (ANOVA) followed by Tukey’s multiple-comparison test. Lowercase letters above the bars indicate statistically significant differences (*p* < 0.05): a, compared with the uninfected control group; b, compared with the *P. berghei* ANKA-infected (negative control) group; c, compared with the chloroquine-treated group; d, compared with the 200 mg/kg *B. balsamifera* ethanolic leaf extract group; f, compared with the 600 mg/kg *B. balsamifera* ethanolic leaf extract group.

Serum liver function parameters were evaluated at the end of the Peters’ four-day suppressive test, and the results are presented in Figure 5. The untreated *P. berghei* ANKA-infected group exhibited the highest mean AST activity (222.20 U/L), which was significantly higher than that of the uninfected control group (92.00 U/L), the chloroquine-treated group, and the 600 mg/kg *B. balsamifera* extract-treated group (*p* < 0.05). Mean AST activities in mice treated with 200, 400, and 600 mg/kg of the extract were 190.20, 147.60, and 120.60 U/L, respectively. Although a numerical decrease in AST activity was observed with increasing extract dose, only the 600 mg/kg group showed a statistically significant reduction compared with the untreated infected group (*p* < 0.05). The untreated infected group also exhibited the highest mean ALT activity (72.20 U/L), whereas mean ALT activities were 58.40, 48.20, and 32.60 U/L in the 200, 400, and 600 mg/kg extract-treated groups, respectively. However, no statistically significant differences in ALT activity were detected among the experimental groups (*p* > 0.05). ALP activity also did not differ significantly among the experimental groups (*p* > 0.05), although the 200 mg/kg extract-treated group showed a numerically higher mean ALP activity than the other extract-treated groups.

### 3.10. Effect of B. balsamifera Leaf Extract on Liver Histopathology in the Four-Day Suppressive Test

**Figure 6:**
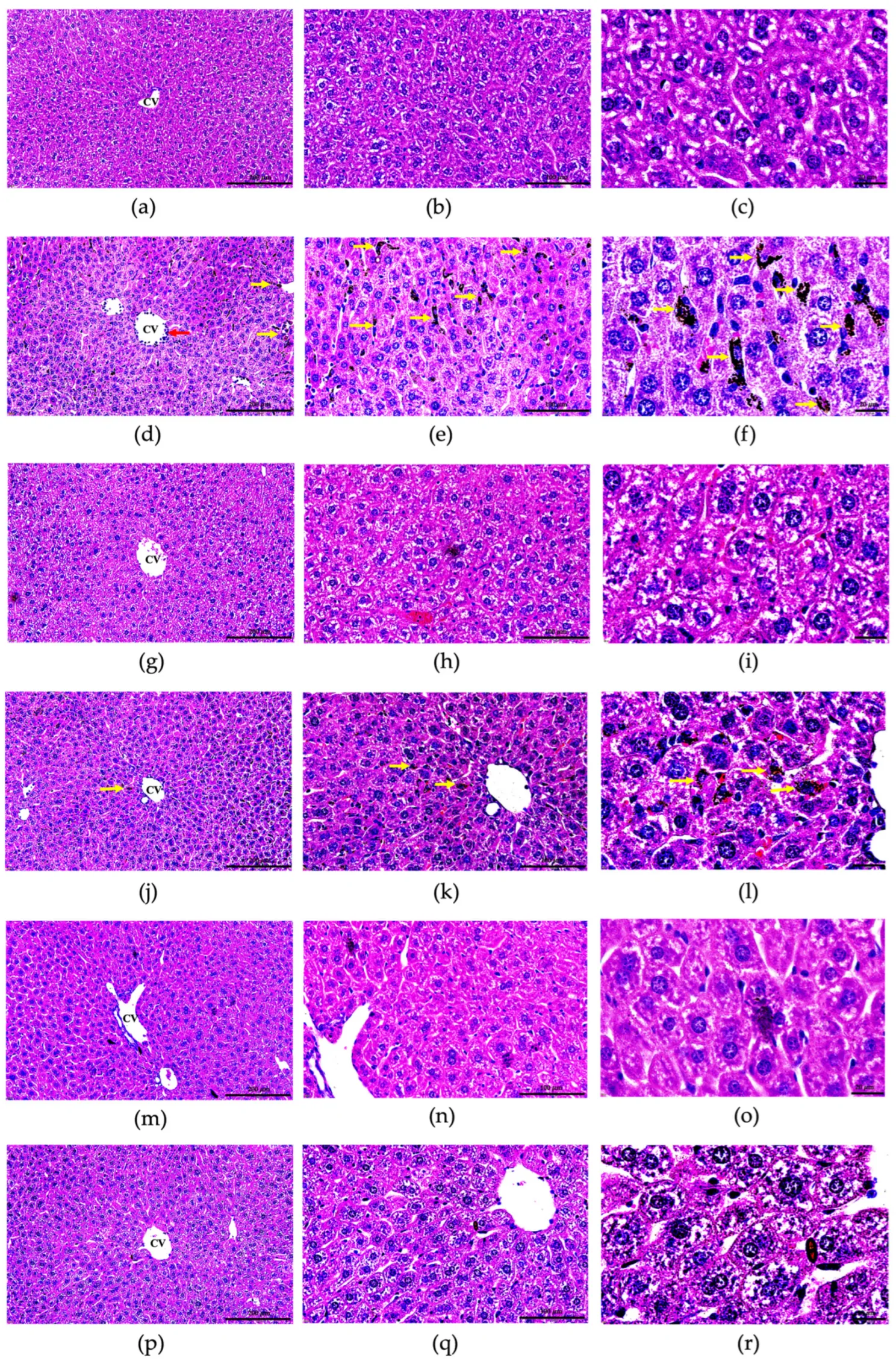
Histopathological changes in liver sections of *P. berghei* ANKA-infected mice following treatment with the ethanolic leaf extract of *B. balsamifera* (hematoxylin and eosin staining). Panels represent the following groups: (**a**–**c**) uninfected control; (**d**–**f**) untreated *P. berghei* ANKA-infected control; (**g**–**i**) chloroquine-treated group (25 mg/kg); (**j**–**l**) *B. balsamifera* extract-treated group (200 mg/kg); (**m**–**o**) extract-treated group (400 mg/kg); and (**p**–**r**) extract-treated group (600 mg/kg). Images in the first column (**a**,**d**,**g**,**j**,**m**,**p**), second column (**b**,**e**,**h**,**k**,**n**,**q**), and third column (**c**,**f**,**i**,**l**,**o**,**r**) were captured at ×20, ×40, and ×100 magnification, respectively. Scale bars: 200 μm (×20), 100 μm (×40), and 20 μm (×100). Red arrows indicate inflammatory cell infiltration, whereas yellow arrows indicate parasitized erythrocytes and malaria pigment deposition, visible as small brown punctate deposits.

Figure 6 shows the histological characteristics of liver tissues from all experimental groups. Liver sections from the uninfected control group (Figure 6a–c) showed normal hepatic architecture, with intact portal veins, bile canaliculi, and regularly arranged hepatocytes. In contrast, liver tissues from the untreated *P. berghei* ANKA-infected group (Figure 6d–f) exhibited prominent histopathological alterations associated with malaria infection, including extensive inflammatory cell infiltration, sinusoidal dilation, and hypertrophy of Kupffer cells containing abundant hemozoin pigment. Administration of the ethanolic leaf extract of *B. balsamifera* at 200 mg/kg (Figure 6j–l) resulted in partial improvement of hepatic morphology, with reduced inflammatory cell infiltration and decreased hemozoin deposition compared with the untreated infected group; however, some histopathological alterations remained evident. Treatment with higher doses of 400 mg/kg (Figure 6m–o) and 600 mg/kg (Figure 6p–r) was associated with greater preservation of hepatic architecture, characterized by reduced inflammatory cell infiltration, decreased hemozoin accumulation, and improved overall tissue morphology. The chloroquine-treated group (25 mg/kg; Figure 6g–i) showed substantial preservation of normal hepatic histological features, with minimal inflammatory changes and reduced hemozoin deposition compared with the untreated infected group.

### 3.11. Effect of B. balsamifera Leaf Extract on Spleen Histopathology in the Four-Day Suppressive Test

**Figure 7:**
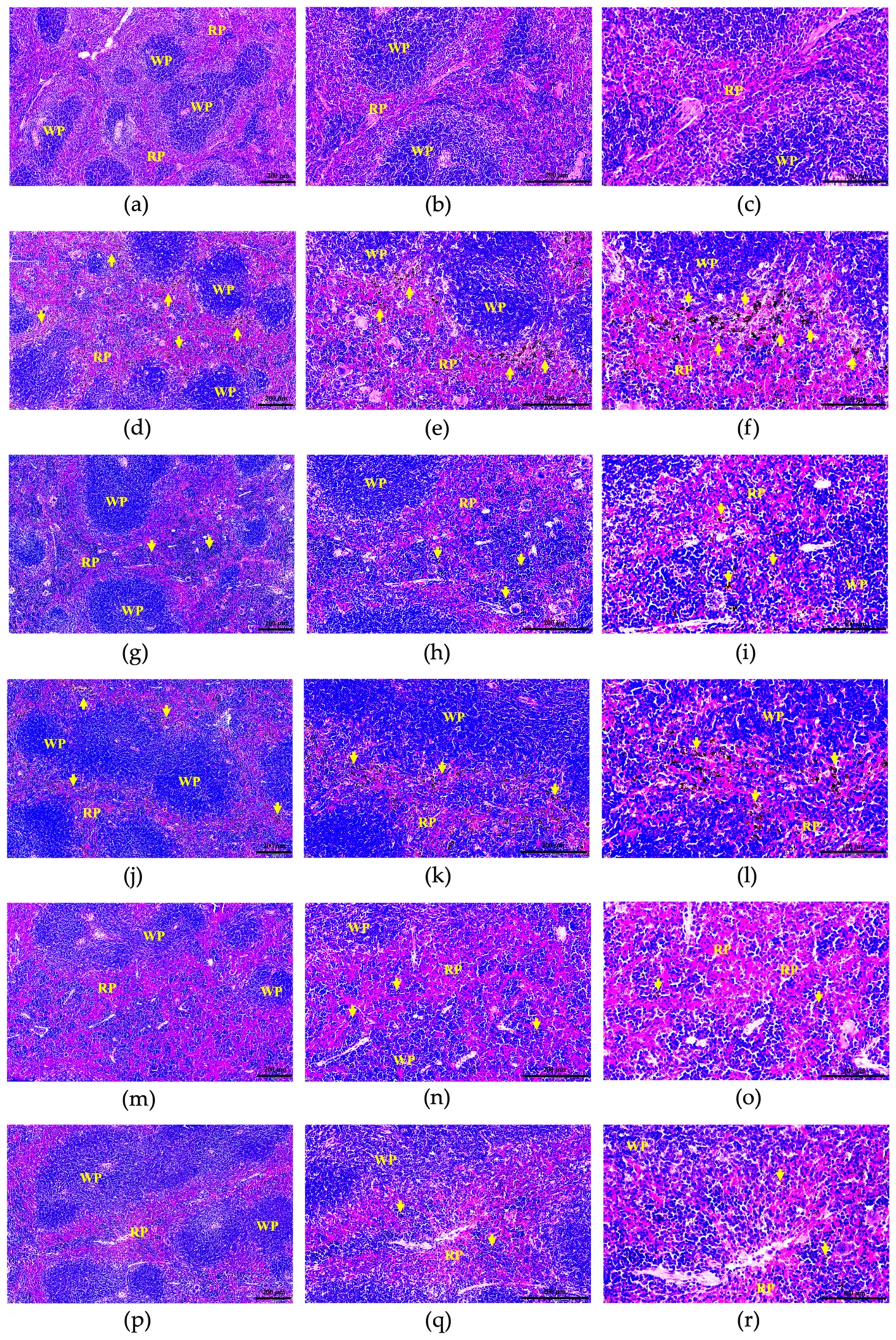
Histopathological changes in spleen sections of *P. berghei* ANKA-infected mice following treatment with the ethanolic leaf extract of *B. balsamifera* (hematoxylin and eosin staining). Panels represent the following groups: (**a**–**c**) uninfected control; (**d**–**f**) untreated *P. berghei* ANKA-infected control; (**g**–**i**) chloroquine-treated group (25 mg/kg); (**j**–**l**) *B. balsamifera* extract-treated group (200 mg/kg); (**m**–**o**) extract-treated group (400 mg/kg); and (**p**–**r**) extract-treated group (600 mg/kg). Images in the first column (**a**,**d**,**g**,**j**,**m**,**p**), second column (**b**,**e**,**h**,**k**,**n**,**q**), and third column (**c**,**f**,**i**,**l**,**o**,**r**) were captured at ×10, ×20, and ×40 magnification, respectively. Scale bars: 200 μm (×10), 200 μm (×20), and 100 μm (×40). Yellow arrows indicate areas of congestion. WP, white pulp; RP, red pulp.

The effects of *P. berghei* ANKA infection and treatment with the ethanolic leaf extract of *B. balsamifera* on splenic histological organization are presented in Figure 7. The uninfected control group (Figure 7a–c) showed normal splenic architecture, characterized by well-defined white pulp and red pulp regions. In contrast, the untreated *P. berghei* ANKA-infected group (Figure 7d–f) exhibited marked alterations in splenic morphology, including disruption of white and red pulp organization, extensive congestion of the red pulp with erythrocytes and parasitized erythrocytes, and accumulation of malaria pigment within pulp histiocytes and sinusoidal lining cells. Treatment with the ethanolic leaf extract of *B. balsamifera* was associated with improved splenic architecture. At 200 mg/kg (Figure 7j–l), partial restoration of tissue organization was observed, although some structural abnormalities remained. Higher doses of 400 mg/kg (Figure 7m–o) and 600 mg/kg (Figure 7p–r) were associated with greater preservation of splenic compartmentalization, accompanied by reduced congestion and decreased malaria pigment accumulation. The chloroquine-treated group (25 mg/kg; Figure 7g–i) exhibited substantial preservation of normal splenic morphology compared with the untreated infected group.

### 3.12. Effect of B. balsamifera Leaf Extract on Body Weight Changes in the Four-Day Suppressive Test

Changes in body weight were monitored throughout the Peters’ four-day suppressive test in *P. berghei* ANKA-infected mice, and the results are presented in Table 6. No significant differences in body weight were observed among the experimental groups at day 0 (*p* > 0.05). Following infection, the untreated *P. berghei* ANKA-infected group exhibited a reduction in body weight, with an average change of −3.94 ± 3.48% from baseline. In contrast, the chloroquine-treated group showed an increase in body weight by day 4 (2.21 ± 1.49%). Treatment with the ethanolic leaf extract of *B. balsamifera* at doses of 200 and 400 mg/kg resulted in minimal changes in body weight, with mean changes of 0.48 ± 2.74% and 0.89 ± 2.12%, respectively. The 600 mg/kg treatment group showed a greater increase in body weight (2.72 ± 2.51%) than the lower-dose extract groups. Mice treated with 600 mg/kg of the ethanolic leaf extract exhibited a significantly greater change in body weight than the untreated infected group (*p* < 0.05). These findings suggest that treatment with the ethanolic leaf extract attenuated infection-associated body weight loss during the experimental period.

### 3.13. Assessment of Clinical Signs, Behavioral Changes, Food and Water Intake, and Body Weight Following Acute Oral Administration of B. balsamifera Leaf Extract

During the 14-day observation period following a single oral administration of *B. balsamifera* ethanolic leaf extract at 2000 mg/kg body weight, no treatment-related adverse clinical signs or behavioral abnormalities were observed in any of the animals. Daily monitoring revealed no signs of acute toxicity, including tremors, muscle rigidity, ataxia, piloerection, abnormal posture, altered grooming behavior, changes in sleep patterns, diarrhea, or other observable physiological disturbances. No mortality was observed during the first 24 h after administration or throughout the 14-day observation period.

Food and water consumption were monitored throughout the study period. No significant differences in food consumption or water intake were observed among the experimental groups (*p* > 0.05) (Table 7). These findings indicate that oral administration of *B. balsamifera* leaf extract did not significantly affect food consumption or water intake under the experimental conditions.

Changes in body weight were assessed on Days 0 and 14 following extract administration. No significant differences in percentage body weight change were observed among the experimental groups (*p* > 0.05) (Table 8). All animals exhibited a progressive increase in body weight throughout the 14-day observation period.

### 3.14. Liver and Kidney Function Biochemical Parameters

Serum biochemical parameters associated with hepatic and renal function were evaluated at the end of the 14-day acute oral toxicity study, and the results are presented in Table 9. Assessment of hepatic function markers, including ALT and ALP, revealed no significant differences between mice treated with a single oral dose of *B. balsamifera* ethanolic leaf extract (2000 mg/kg body weight) and the control groups (*p* > 0.05). Although AST levels were slightly higher in the normal control group than in the vehicle control and extract-treated groups, the AST values in all groups remained within the expected physiological range, with no corresponding treatment-related clinical or histopathological abnormalities observed. ALT and ALP levels were comparable among the experimental groups. Similarly, renal function markers, including BUN and serum creatinine, did not differ significantly among the experimental groups (*p* > 0.05). Overall, the absence of significant changes in hepatic and renal biochemical parameters suggests that a single oral administration of *B. balsamifera* ethanolic leaf extract at 2000 mg/kg did not produce apparent hepatic or renal toxicity under the conditions of this acute toxicity study.

### 3.15. Histopathological Examination of Liver and Kidney Tissues Following Acute Oral Toxicity Evaluation

#### 3.15.1. Liver Histopathology

**Figure 8:**
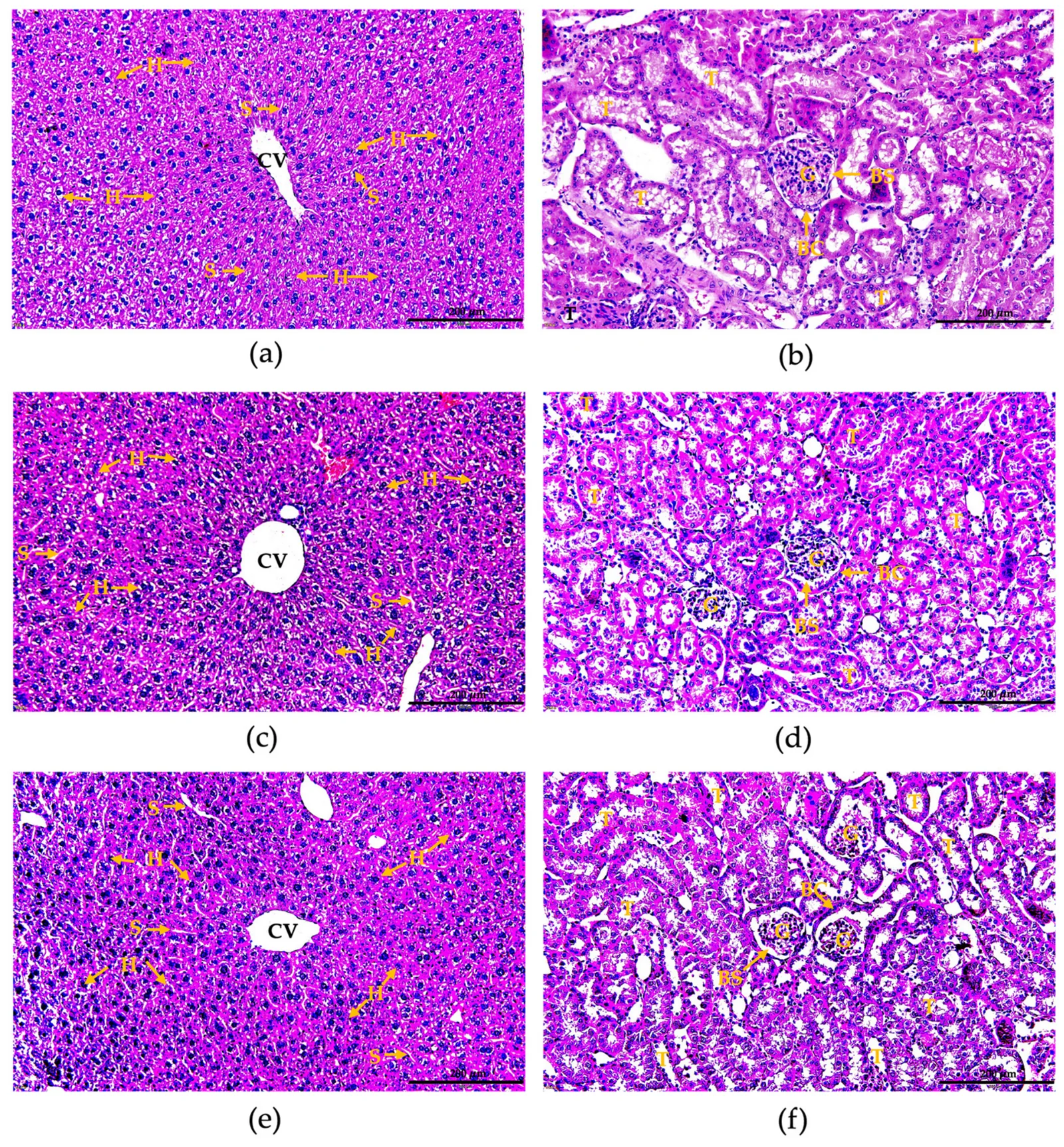
Histopathological evaluation of liver and kidney tissues following acute oral administration of *B. balsamifera* ethanolic leaf extract. Representative liver and kidney sections from the normal control group are shown in panels (**a**,**b**), respectively; sections from mice administered 7% Tween 80 (vehicle control) are shown in panels (**c**,**d**); and sections from mice treated with *B. balsamifera* ethanolic leaf extract at 2000 mg/kg body weight are shown in panels (**e**,**f**). Tissue sections were stained with hematoxylin and eosin (H&E) and examined at ×20 magnification. Scale bar = 200 μm. Yellow arrow indicate liver and renal structure. CV, central vein; H, hepatocyte; S, sinusoidal capillary; G, glomerulus; BC, Bowman’s capsule; BS, Bowman’s space; T, renal tubule.

Representative liver sections from the control, vehicle-treated, and *B. balsamifera* ethanolic leaf extract-treated groups (2000 mg/kg) are shown in Figure 8. The liver tissues of the normal control group (Figure 8a) exhibited normal hepatic architecture, characterized by radially arranged hepatocyte cords surrounding the central vein and regularly distributed hepatic sinusoids. Hepatocytes showed distinct cellular boundaries, eosinophilic cytoplasm, and centrally located nuclei, with no observable histopathological abnormalities.

Similarly, liver sections from the vehicle-treated group (Figure 8c) showed preserved hepatic architecture, with normal hepatocyte arrangement and intact sinusoidal structures. No evidence of hepatocellular degeneration, inflammatory cell infiltration, vascular alterations, or tissue damage was observed. The *B. balsamifera* ethanolic leaf extract-treated group (2000 mg/kg; Figure 8e) also exhibited preserved hepatic architecture without apparent histopathological abnormalities. The hepatic parenchyma remained intact, with no detectable lesions, including hepatocyte degeneration, necrosis, inflammatory infiltration, sinusoidal distortion, or vascular congestion.

#### 3.15.2. Kidney Histopathology

Representative kidney sections from the control, vehicle-treated, and *B. balsamifera* ethanolic leaf extract-treated groups are presented in Figure 8. The kidneys of the normal control group (Figure 8b) showed normal renal architecture, including well-defined glomeruli, preserved Bowman’s spaces, and regularly arranged renal tubules. The tubular epithelium remained intact, with no evidence of degeneration or structural abnormalities. The vehicle-treated group (Figure 8d) exhibited comparable renal morphology, with preserved cortical organization and normal glomerular and tubular structures. No inflammatory cell infiltration or interstitial abnormalities were observed. Kidney sections from mice treated with *B. balsamifera* ethanolic leaf extract at 2000 mg/kg (Figure 8f) showed preserved renal architecture, with intact glomeruli, Bowman’s capsules, and renal tubules. No microscopic alterations, including tubular degeneration, necrosis, inflammatory cell infiltration, fibrosis, or structural distortion, were detected.

## 4. Discussion

Natural products continue to represent a valuable reservoir for pharmacological discovery due to their extensive structural diversity and long-standing ethnopharmacological applications [31]. In the present study, the antiplasmodial potential of *B. alsamifera* leaf extracts was systematically evaluated through an integrated approach involving in vitro antiplasmodial activity, cytotoxicity assessment, phytochemical characterization, in vivo antiplasmodial assessment, and toxicity evaluation. Among the tested preparations, the ethanolic leaf extract demonstrated strong in vitro antiplasmodial activity against the chloroquine-resistant *P. falciparum* K1 strain, with an IC_50_ value of 2.65 µg/mL, whereas the aqueous extract exhibited substantially weaker activity (IC_50_ = 84.54 µg/mL). According to established criteria for plant-derived antimalarial agents, the ethanolic extract can be classified as highly active, whereas the aqueous extract shows weak activity. The observed activity of the ethanolic extract is comparable to previous reports describing potent antiplasmodial effects of ethanolic extracts from Thai medicinal plants and traditional herbal formulations against *P. falciparum* K1, with IC_50_ values ranging from 2.81 to 15.5 µg/mL when evaluated using the pLDH assay [32]. The marked difference in antiplasmodial activity between ethanolic and aqueous extracts highlights the important influence of extraction solvent on the recovery of bioactive metabolites. Ethanol is generally more efficient than water for extracting moderately polar secondary metabolites, including flavonoids, phenolic compounds, terpenoids, and alkaloid derivatives, which have been frequently associated with antiplasmodial properties [32,33]. In contrast, aqueous extraction may preferentially recover highly polar constituents that may exhibit lower antiplasmodial activity. The strong activity of the ethanolic extract against the chloroquine-resistant *P. falciparum* K1 strain is particularly noteworthy, and suggests that the extract contains bioactive constituents capable of inhibiting parasite growth in this resistant strain. However, further mechanistic investigations are required to determine whether these compounds act through pathways distinct from classical resistance-associated mechanisms, including alterations in drug transport and parasite metabolic processes.

In addition to its in vitro activity, the ethanolic extract of *B. balsamifera* demonstrated significant in vivo antiplasmodial activity in *P. berghei* ANKA-infected mice using the Peters’ 4-day suppressive test. Administration of the extract resulted in a dose-dependent reduction in parasitemia, with suppression rates of 42.29%, 70.74%, and 76.54% at doses of 200, 400, and 600 mg/kg, respectively. Although parasite suppression increased with increasing dose, the difference between the 400 and 600 mg/kg groups was not statistically significant, suggesting that the antimalarial effect may have approached a plateau at higher doses. These findings indicate that the ethanolic extract contains bioactive constituents that may contribute to the observed in vivo antiplasmodial effects. Similar dose-dependent responses have been reported for several medicinal plants native to Southeast Asia, in which crude ethanolic extracts demonstrated inhibitory activity against rodent malaria parasites using comparable experimental models. These findings support the potential of *B. balsamifera* as a source of antimalarial bioactive compounds and warrant further investigation to identify and characterize the constituents responsible for its activity.

Although the ethanolic extract exhibited significant parasite suppression, its efficacy remained lower than that of chloroquine, which achieved 98.01% suppression at 25 mg/kg. The lower efficacy of the crude extract compared with the standard drug may be attributable to several factors, including the relatively low concentration of individual active constituents in the crude preparation, as well as differences in bioavailability, absorption, metabolism, and pharmacokinetic properties. Nevertheless, parasite suppression exceeding 50% was observed at doses of 400 and 600 mg/kg, indicating moderate antiplasmodial activity according to commonly applied criteria for evaluating plant extracts in rodent malaria models. Compared with previously reported medicinal plants, the ethanolic extract of *B. balsamifera* showed higher parasite suppression than that reported for *B. abyssinica* leaves (49.51%) [34], *O. lamiifolium* leaves (35.53%) [35], *M. salicifolia* roots (59.13%) [36], and *V. amygdalina* leaves (60.78%) [37]. These findings suggest that *B. balsamifera* possesses promising antiplasmodial activity in *P. berghei* ANKA-infected mice, particularly at doses of 400 and 600 mg/kg. Although parasite suppression increased numerically with increasing dose, the difference between the 400 and 600 mg/kg groups was not statistically significant, suggesting that the antiplasmodial effect may have approached a plateau at the higher dose. This observation is consistent with previous reports of dose-related antiplasmodial activity in other medicinal plants [38]. The observed activity may be associated with the diverse secondary metabolites present in the extract, including alkaloids, terpenoids, flavonoids, xanthones, anthraquinones, phenolic compounds, and sesquiterpenes, many of which have been reported to possess antiplasmodial properties [39]. In the present study, the antiplasmodial effects may therefore result from the combined contributions of multiple phytochemical constituents, particularly flavonoids, alkaloids, and terpenoid-related compounds. These constituents may interfere with parasite survival and development through multiple mechanisms, although further studies are required to determine their individual or combined contributions to the antiplasmodial activity of *B. balsamifera* [40].

Assessment of cytotoxicity demonstrated that the ethanolic leaves extract of *B. balsamifera* exhibited a CC_50_ value of 61.03 µg/mL against Vero cells. Based on established cytotoxicity classification criteria for crude plant extracts, samples with CC_50_ values < 1 µg/mL, 1–10 µg/mL, 10–30 µg/mL, and >30 µg/mL are classified as highly toxic, moderately toxic, mildly toxic, and non-toxic, respectively [27]. The CC_50_ value observed for the ethanolic extract exceeded the National Cancer Institute (NCI) threshold of 30 µg/mL for crude plant extracts, indicating low cytotoxicity toward mammalian cells under the tested conditions. Furthermore, the extract exhibited a SI of 23.03, calculated from the ratio of CC_50_ to its antiplasmodial concentration (IC_50_), indicating preferential activity against *P. falciparum* relative to Vero cells. An SI value ≥ 10 is frequently used as an indicator of promising selectivity in antiplasmodial screening [25]. Therefore, the relatively high SI observed suggests that the antiplasmodial activity of the ethanolic extract is not primarily attributable to nonspecific cytotoxicity. These findings support the potential of *B. balsamifera* as a source of antimalarial bioactive compounds. However, further studies involving bioassay-guided fractionation, isolation and characterization of active constituents, mechanistic investigations, and pharmacokinetic evaluation are required to determine their potential for antimalarial drug development.

Phytochemical profiling by LC-QTOF-MS analysis revealed a diverse array of tentatively identified metabolites in the ethanolic leaf extract of *B. balsamifera*, including flavonoids such as taxifolin, naringenin, fisetin, quercetin 3-methyl ether, apigenin, diosmetin, rhamnetin, glycitein, koparin 2′-methyl ether, and quercetin tetramethyl (5,7,3′,4′) ether; the phenolic compound lecanoric acid; terpenoid-related metabolites including confertifoline, α-farnesene, and alantolactone; and the alkaloid mitragynine. Because these compounds were tentatively identified based on LC-QTOF-MS data, their identities should be confirmed by comparison with authentic standards and/or isolation and spectroscopic characterization. Nevertheless, the chemical profile provides evidence of the diverse phytochemical composition of the extract. Flavonoids, alkaloids, terpenoids, and phenolic compounds have been widely investigated for their antiplasmodial properties [41,42]. Therefore, the antiplasmodial activity observed in the present study may be attributable to the combined contributions of multiple constituents rather than a single compound [43].

Several flavonoids detected in the extract have previously been associated with antiplasmodial activity. Fisetin has been reported to inhibit parasite proteases involved in hemoglobin degradation, including falcipain-2 and plasmepsin II, which may interfere with parasite nutrient acquisition and intraerythrocytic development [44]. Quercetin has also demonstrated antiplasmodial activity through interactions with multiple parasite targets, including calcium-dependent protein kinase 4 (*Pf*CDPK4) and cyclic guanosine monophosphate-dependent protein kinase (*Pf*PKG), which are involved in essential processes during parasite development [45]. In *P. berghei*-infected mice, quercetin has additionally been reported to modulate inflammatory responses, including reductions in TNF-α and IFN-γ and increases in IL-10 and IL-4, suggesting potential immunomodulatory effects during malaria infection [46]. Similarly, apigenin has been reported to inhibit parasite growth through mechanisms involving calcium homeostasis and heme detoxification, including inhibition of β-hematin formation [47]. Luteolin, although not detected in the positive-ionization LC-QTOF-MS analysis reported in Table 3, was identified in the negative ionization mode (Table 2) and has previously demonstrated activity against both chloroquine-sensitive *P. falciparum* 3D7 and chloroquine-resistant 7G8 strains [48]. Computational studies have further suggested potential interactions between luteolin and *P. falciparum* dihydrofolate reductase-thymidylate synthase (*Pf*DHFR-TS) [49].

Taken together, these findings suggest that the diverse phytochemical composition of *B. balsamifera* may contribute to its antiplasmodial activity through multiple biological pathways, including interference with parasite proteases, kinase signaling, heme detoxification, and host inflammatory responses [50,51,52]. However, the present study did not directly establish the contribution or mechanism of action of individual compounds. Therefore, the observed antiplasmodial activity should be interpreted as a potential consequence of the combined activity of multiple constituents rather than confirmed synergistic or multitarget effects. Further bioassay-guided fractionation, isolation and characterization of active constituents, and mechanistic studies are required to determine which compounds contribute most substantially to the antiplasmodial activity of *B. balsamifera*.

Severe hematological alterations are common complications of malaria and may result from destruction of infected erythrocytes, increased clearance of uninfected erythrocytes, impaired erythropoiesis, and malaria-associated inflammatory responses [53]. In this study, *P. berghei* ANKA infection was associated with significant alterations in selected hematological parameters. The untreated infected mice exhibited a significantly lower RBC count than the uninfected controls, whereas no statistically significant differences in Hb concentration or HCT were observed among the experimental groups. Therefore, the observed reductions in Hb and HCT should be interpreted cautiously and do not provide sufficient evidence of marked anemia within the experimental period. In contrast, more pronounced changes were observed in WBC and platelet counts. The untreated infected group exhibited a markedly elevated WBC count, whereas treatment with chloroquine or *B. balsamifera* extract was associated with significantly lower WBC counts. This finding may reflect attenuation of the infection-associated leukocyte response, although inflammatory mediators were not directly assessed in the present study [54,55,56]. In addition, *P. berghei* infection resulted in a marked reduction in platelet count. Treatment with a higher dose of *B. balsamifera* extract, particularly 400 and 600 mg/kg, was associated with higher platelet counts than those observed in untreated infected mice. However, platelet counts remained below those of the uninfected control group, indicating partial attenuation rather than complete normalization of malaria-associated thrombocytopenia. Similar changes in hematological parameters have been reported for other medicinal plants, including *C. odorata* leaf extract, which was associated with increased RBC counts, Hb concentrations, and HCT values in malaria-infected experimental models [57]. These hematological effects may partly reflect the reduction in parasite burden following extract administration because decreased parasitemia could limit parasite-associated erythrocyte destruction and other infection-related hematological disturbances. The phytochemical constituents identified in *B. balsamifera*, including flavonoids and phenolic compounds, may also contribute through their reported antioxidant and anti-inflammatory properties [58]. However, oxidative stress biomarkers, inflammatory mediators, erythrocyte membrane integrity, and erythropoietic responses were not directly evaluated. Therefore, the mechanisms underlying the observed hematological changes remain speculative and require further investigation.

As well as antiplasmodial activity, *B. balsamifera* extract exhibited protective effects against malaria-associated tissue injury, particularly in the liver and spleen, which are the major organs affected during malaria infection. Histopathological alterations observed in the untreated infected mice included inflammatory cell infiltration, sinusoidal dilation, and hemozoin accumulation in the liver, consistent with previous reports of malaria-associated hepatic injury [59]. Similarly, splenic enlargement, red pulp congestion, disruption of splenic architecture, and malaria pigment accumulation were observed, reflecting pathological changes associated with malaria infection [60]. Treatment with *B. balsamifera* extract attenuated these histopathological alterations in a dose-dependent manner, with greater improvement observed at 400 and 600 mg/kg, suggesting a potential protective effect against malaria-associated tissue injury. Although the precise mechanisms underlying these protective effects remain to be elucidated, they may be associated with the phytochemical constituents of the extract, including flavonoids, phenolic compounds, tannins, and other antioxidant constituents [17]. These compounds have been reported to possess antioxidant and anti-inflammatory activities that may help reduce reactive oxygen species (ROS)-mediated tissue damage and inflammatory responses. Flavonoids, in particular, can scavenge ROS, enhance endogenous antioxidant defenses, and modulate oxidative stress-related inflammatory pathways [61]. Alkaloids may also contribute to tissue protection through modulation of oxidative stress and inflammatory signaling pathways, including Nrf2-related pathways [62]. Therefore, the antioxidant and anti-inflammatory properties of the phytochemicals present in *B. balsamifera* may contribute to the preservation of hepatic and splenic architecture observed in treated mice.

Consistent with the pathophysiology of malaria, alterations in serum liver enzyme activities were observed in *P. berghei* ANKA-infected mice. In particular, the untreated infected group exhibited the highest AST and ALT activities, with AST activity being significantly higher than that in the uninfected control group. These changes may reflect malaria-associated hepatic injury resulting from parasite infection, inflammatory responses, and oxidative stress [28]. However, no statistically significant differences in ALT or ALP activities were detected among the experimental groups. Therefore, the numerical changes in these parameters should be interpreted cautiously and should not be considered definitive evidence of treatment-related improvement in hepatic function. Treatment with *B. balsamifera* ethanolic leaf extract was associated with a numerical decline in AST and ALT activities with increasing dose. However, only the reduction in AST activity at 600 mg/kg reached statistical significance compared with the untreated infected group. Thus, the present findings provide evidence of a parameter-specific biochemical effect rather than a generalized hepatoprotective effect. Although the mean ALT activity at 600 mg/kg was lower than that in the untreated infected group, this difference was not statistically significant and should be regarded as a numerical trend rather than a confirmed treatment effect. Moreover, because AST is not specific to the liver, its reduction alone is insufficient to establish direct hepatoprotection. The observed reduction in AST activity may be related, at least in part, to the phytochemical composition of *B. balsamifera*. The flavonoids, phenolic compounds, and alkaloids identified in the extract have been reported to possess antioxidant and cytoprotective properties [18,63]. These constituents could potentially contribute to limiting oxidative and inflammatory tissue damage during malaria infection [64]. Nevertheless, oxidative stress biomarkers, antioxidant enzyme activities, and molecular indicators of hepatic injury were not directly assessed in the present study; therefore, this mechanism remains speculative. Akinyemi et al. reported that treatment with ethanolic leaf extract of *B. sapida* at 400 mg/kg significantly reduced AST and ALT activities in malaria-infected animals, an effect attributed to the antioxidant properties of the extract and stabilization of cellular membranes [64]. Although this finding provides relevant context, the present results should be interpreted more cautiously because significant treatment-associated changes were observed only for AST. The reduction in AST activity may also be secondary to decreased parasitemia and an improvement in the overall physiological condition of infected animals rather than a direct hepatoprotective action of the extract. Further studies incorporating oxidative stress biomarkers, antioxidant enzyme activities, liver-specific injury markers, and histopathological or molecular endpoints are therefore required to determine whether *B. balsamifera* exerts a direct protective effect on hepatic tissue.

In this study, we evaluated the acute oral toxicity of the ethanolic leaf extract of *B. balsamifera* in ICR mice. No mortality or treatment-related signs of acute toxicity were observed following a single oral administration of the extract at the limit dose of 2000 mg/kg body weight during the 14-day observation period. These findings indicate that the median lethal dose (LD_50_) of the extract is likely to be greater than 2000 mg/kg under the conditions of the present study. According to the relevant acute oral toxicity classification criteria, the extract showed a relatively low acute toxicity at the tested dose. The absence of treatment-related abnormalities in clinical signs, food and water consumption, and body weight further supports the favorable acute safety profile of the extract. The present findings are consistent with a previous study [65], which also reported an LD_50_ value of >2000 mg/kg for *B. balsamifera* extract. Differences in reported toxicity outcomes among studies may be attributable to variations in extraction procedures, dose selection, administration protocols, and experimental conditions, including animal species, age, sex, and body weight. For example, Pang et al. reported minor hepatic alterations, including hepatocellular vacuolar changes and increased Kupffer cell numbers, in rats administered *B. balsamifera* extract at 100% *w*/*v* [16]. The discrepancy between these findings and the absence of hepatic histopathological abnormalities in the present study may reflect differences in the experimental design, extract concentration, animal characteristics, and dosing procedures. Therefore, direct comparison between studies should be made cautiously. In the present study, the absence of histopathological alterations in the liver and kidney, together with the lack of significant changes in serum ALT, ALP, BUN, and creatinine levels, provides additional evidence supporting the absence of apparent acute toxicity at 2000 mg/kg.

Food and water consumption are important indicators for evaluating the general health status and potential toxic effects in experimental animals [66]. In the present study, mice treated with *B. balsamifera* ethanolic leaf extract at 2000 mg/kg showed slightly lower food and water consumption than the control groups; however, these differences were not statistically significant. The observed values were also comparable to those reported previously for healthy mice [67]. The absence of significant changes in food and water consumption suggests that acute administration of the extract did not adversely affect feeding or drinking behavior under the conditions of the present study. Together with the absence of treatment-related clinical signs and mortality during the 14-day observation period, these findings further support the favorable acute tolerability of the extract.

A body weight loss of more than 10% from the initial body weight following exposure to a test substance is commonly considered an important indicator of potential toxicity [30]. In the present study, body weight increased progressively in both the control and *B. balsamifera*-treated groups during the 14-day observation period, with no significant differences in body weight or percentage body weight change among the groups. These findings are consistent with previous studies reporting no significant treatment-related changes in body weight following administration of *B. balsamifera* or other medicinal plant extracts [68,69]. Although the extract-treated mice showed a numerically lower percentage increase in body weight than the control groups, the difference was not statistically significant, indicating that this variation was unlikely to be treatment-related. Furthermore, the absence of significant changes in food and water consumption, together with the lack of treatment-related abnormalities in organ weights, serum biochemical parameters, and liver and kidney histopathology, supports the absence of an apparent adverse effect of the extract on general growth and body weight during the observation period.

Among the physiological systems evaluated, the hematopoietic system is particularly sensitive to toxic insults. Therefore, hematological parameters are widely used as biomarkers for assessing systemic toxicity in experimental animals [70]. The liver and kidneys are also important target organs in toxicity assessment because of their central roles in the metabolism, detoxification, and elimination of xenobiotics [71]. Hepatic toxicity is commonly evaluated using serum enzymes such as AST, ALT, and ALP, whereas renal function is assessed using parameters such as BUN and creatinine [72,73]. In the present study, serum ALT, ALP, BUN, and creatinine levels did not differ significantly between the *B. balsamifera*-treated and control groups, indicating no apparent adverse effects on these biochemical indicators following acute administration of the extract at 2000 mg/kg. Although the toxicity of *B. balsamifera* leaf extracts has been investigated, a safety assessment was considered necessary for the extract used in the present investigation. The plant material used in this study was obtained from Southern Thailand; it was taxonomically authenticated, and deposited as a voucher specimen (SMD072012003). Because the phytochemical composition of crude botanical extracts may vary according to geographical origin, growing and storage conditions, and extraction procedures, previously reported toxicity findings may not be fully transferable to a different extract preparation. In addition, we used ICR mice and evaluated the same extract subsequently administered in the antimalarial experiments at doses of 200–600 mg/kg, thereby providing a safety margin under the experimental conditions of the present study. The absence of major biochemical abnormalities observed in this study is consistent with previous reports showing no significant adverse effects on hepatic or renal biochemical parameters following administration of *B. balsamifera* extracts at doses of 1000, 3000, and 5000 mg/kg [65]. Thus, the present findings should be regarded as complementary to, rather than merely a replication of, the previously reported safety data. Interestingly, serum AST activity was significantly lower in the extract-treated group than in the normal control group. However, AST is not specific to the liver and may also originate from other tissues; therefore, this difference should not be interpreted as evidence of a hepatotoxic or hepatoprotective effect. Importantly, AST activity was not elevated following administration of the extract, and no treatment-related abnormalities were observed in liver histopathology. Taken together, the biochemical and histopathological findings provide no evidence of apparent hepatic or renal toxicity following a single oral administration of *B. balsamifera* extract at 2000 mg/kg under the conditions of the present study.

This study has several limitations. The antimalarial efficacy was evaluated using a short-term four-day suppressive test; therefore, the findings primarily reflect early suppressive activity and do not provide information on longer-term therapeutic outcomes or parasite recrudescence. In addition, although antioxidant and anti-inflammatory mechanisms may contribute to the observed biological effects, these mechanisms were not directly evaluated using specific molecular or biochemical biomarkers. Further studies involving isolation and characterization of active constituents and mechanistic investigations are therefore warranted to better clarify the antimalarial activity of *B. balsamifera*.

## 5. Conclusions

The ethanolic leaf extract of *B. balsamifera* demonstrated strong in vitro antiplasmodial activity against chloroquine-resistant *P. falciparum* K1 and dose-dependent in vivo antiplasmodial activity against *P. berghei* ANKA, achieving 76.54% suppression at 600 mg/kg. Treatment improved malaria-associated hematological, biochemical, and histopathological alterations, particularly in the liver and spleen. A single oral dose of 2000 mg/kg was well tolerated during the 14-day observation period, with no apparent treatment-related toxicity. These findings support the antiplasmodial potential and preliminary acute safety of *B. balsamifera* leaf extract. Further studies are warranted to identify active constituents and elucidate their pharmacological mechanisms.

## Figures and Tables

**Table 1:** In vitro antiplasmodial activity, cytotoxicity, and selectivity index of *B. balsamifera* leaf extracts.

Crude Extract	IC_50_ (µg/mL)	CC_50_ (µg/mL)	SI
Aqueous extract of *B. balsamifera*	84.54 ± 1.88	>200	>2.36
Ethanolic extract of *B. balsamifera*	2.65 ± 0.44	61.03 ± 2.07	23.03
Artesunate *	8.92 ± 0.001	-	-
Chloroquine	0.48 ± 0.04	-	-
Doxorubicin	-	3.07 ± 0.58	-

Data are presented as the mean  ±  standard error mean (SD) of three independent experiments. IC_50_, half-maximal inhibitory concentration; CC_50_, half-maximal cytotoxic concentration; SI, selectivity index (CC_50_/IC_50_) * Concentration of artesunate with IC_50_ unit expressed in ng/mL.

**Table 2:** Tentatively identified metabolites in the ethanolic leaf extract of *B. balsamifera* by LC-QTOF-MS in negative ionization mode.

No.	*m*/*z*	RT (min)	Identified Compound	Formula	Molecular Weight (g/mol)	Characteristic MS/MS Fragments (*m*/*z*)
1	303.051	15.420	(±)-Taxifolin	C_15_H_12_O_7_	304.058	285.040, 217.050, 125.025, 57.034
2	187.097	16.146	Methyl N-(a-methylbutyryl) glycine	C_9_H_16_O_4_	188.104	187.098, 125.097, 83.049
3	285.041	18.138	Luteolin	C_15_H_10_O_6_	286.048	151.004, 133.030, 65.003
4	301.036	18.464	Quercetin	C_15_H_10_O_7_	302.043	229.051, 151.004, 65.003
5	317.067	19.028	Lecanoric acid	C_16_H_14_O_7_	318.074	289.071, 195.030, 167.035, 65.003
6	269.045	19.792	Apigenin	C_15_H_10_O_5_	270.052	187.040, 151.004, 119.050
7	271.061	19.942	(±)-Naringenin	C_15_H_12_O_5_	272.068	151.004, 119.050
8	299.056	20.130	Diosmetin	C_16_H_12_O_6_	300.063	151.004, 63.024
9	301.072	21.646	Hesperetin	C_16_H_14_O_6_	302.079	135.045, 65.003
10	343.082	23.074	(-)-Usnic acid	C_18_H_16_O_7_	344.089	313.035, 242.022
11	283.061	23.838	Prunetin	C_16_H_12_O_5_	284.068	268.038, 165.019, 117.034
12	299.056	24.076	Diosmetin	C_16_H_12_O_6_	300.063	284.032, 135.045, 63.024
13	313.071	24.402	Koparin 2′-methyl ether	C_17_H_14_O_6_	314.079	298.048, 165.019, 65.003
14	293.212	30.302	13(S)-HOTrE	C_18_H_30_O_3_	294.219	221.153, 113.099
15	293.212	30.603	9-OxoODE	C_18_H_30_O_3_	294.219	221.155, 185.117, 127.042
16	271.228	34.311	2-Hydroxyhexadecanoic acid	C_16_H_32_O_3_	272.235	167.071, 59.014

Abbreviations: *m*/*z*, mass-to-charge ratio; RT, retention time; HOTrE, hydroxyoctadecatrienoic acid; ODE, oxodienoic acid.

**Table 3:** Tentative identification of phytochemical constituents in the ethanolic leaf extract of *B. balsamifera* by LC-QTOF-MS in positive ionization mode.

No.	*m*/*z*	RT (min)	Identified Compound	Formula	Molecular Weight (g/mol)	Characteristic MS/MS Fragments (*m*/*z*)
1	118.086	1.882	Betaine	C_5_H_12_NO_2_	118.087	58.065
2	104.107	1.882	Choline	C_5_H_14_NO	104.107	60.081, 58.065
3	305.065	15.361	(±)-Taxifolin	C_15_H_12_O_7_	304.058	259.060, 231.065, 153.018
4	395.240	16.339	19(R)-hydroxy-PGF1α	C_20_H_36_O_6_	372.251	208.050, 121.051, 81.070
5	377.230	17.240	15-F2t-IsoP	C_20_H_34_O_5_	354.241	153.020, 93.070, 89.060
6	273.075	17.466	(±)-Naringenin	C_15_H_12_O_5_	272.068	149.023, 65.038
7	287.056	18.193	Fisetin	C_15_H_10_O_6_	286.048	153.019, 89.039
8	233.164	18.543	Confertifoline	C_15_H_22_O_2_	232.157	217.159, 133.101, 91.054
9	399.228	18.618	Mitragynine	C_23_H_30_N_2_O_4_	398.220	238.144, 174.091, 110.096
10	317.066	18.856	Quercetin 3-methyl ether	C_16_H_12_O_7_	316.059	301.035, 167.034, 123.044
11	397.212	19.909	Quinine ethyl carbonate	C_23_H_28_N_2_O_4_	396.204	381.181, 281.165, 123.079
12	317.066	20.523	Quercetin 3-methyl ether	C_16_H_12_O_7_	316.058	302.042, 217.049, 153.018, 92.026
13	301.071	21.738	Diosmetin	C_16_H_12_O_6_	300.064	258.052, 163.039
14	391.209	21.775	15(R),19(R)-hydroxy Prostaglandin E2	C_20_H_32_O_6_	368.220	273.146, 121.101
15	317.066	22.026	Rhamnetin	C_16_H_12_O_7_	316.058	271.060, 179.034, 123.044
16	233.153	22.765	Alantolactone	C_15_H_20_O_2_	232.146	173.096, 91.054, 69.070
17	361.235	23.329	11-deoxy-PGE1	C_20_H_34_O_4_	338.246	251.170, 81.069
18	285.075	23.805	Glycitein	C_16_H_12_O_5_	284.068	242.057, 167.034, 55.054
19	318.300	23.905	Phytosphingosine	C_18_H_39_NO_3_	317.293	282.279, 167.033, 60.044
20	315.087	24.156	Koparin 2′-methyl ether	C_17_H_14_O_6_	314.079	272.068, 177.055, 60.044
21	373.199	25.935	Lipoxin A5	C_20_H_30_O_5_	350.210	273.146, 139.006
22	233.153	25.972	Alantolactone	C_15_H_20_O_2_	232.146	135.085, 69.070
23	375.214	26.461	5(S),6(R)-Lipoxin A4	C_20_H_32_O_5_	352.225	273.146, 215.143, 139.006
24	373.198	27.288	Prostaglandin D3	C_20_H_30_O_5_	350.209	273.146, 187.148, 123.041
25	359.113	27.538	Quercetin tetramethyl (5,7,3′,4′) ether	C_19_H_18_O_7_	358.105	301.071, 167.034
26	343.224	29.405	16(R)-hydroxyeicosatetraenoic acid	C_20_H_32_O_3_	320.235	201.126, 83.049
27	399.214	30.294	Resolvin D2	C_22_H_32_O_5_	376.225	283.262, 185.079
28	205.195	31.396	α-Farnesene	C_15_H_24_	204.187	123.117, 95.086
29	467.255	35.681	AL 8810 isopropyl ester	C_27_H_37_FO_4_	444.266	407.198, 287.139, 89.058
30	282.278	35.781	Oleamide	C_18_H_35_NO	281.271	247.242, 83.085, 69.070

Abbreviations: *m*/*z*, mass-to-charge ratio; RT, retention time.

**Table 4:** In vivo antiplasmodial activity of the ethanolic leaf extract of *B. balsamifera* in *P. berghei* ANKA-infected mice.

Group	Treatment Dose (mg/kg)	%Parasitemia	%Suppression
*P. berghei ANKA*-infected	-	40.34 ± 5.16 ^b,c,d,e^	-
Chloroquine	25	0.80 ± 0.27 ^a,c,d,e^	98.01 ± 0.68 ^c,d,e^
*B. balsamifera* ethanolic leaf extract	200	23.28 ± 0.94 ^a,b,d,e^	42.29 ± 2.33 ^b,d,e^
	400	11.80 ± 0.49 ^a,b,c^	70.74 ± 1.22 ^b,c,e^
	600	9.46 ± 0.97 ^a,b,c^	76.54 ± 2.41 ^b,c,d^

Data are presented as mean ± SD (n = 5 mice per group). ^a^ Compared to *Pb*A-infected, ^b^ compared to standard chloroquine, ^c^ compared to 200 mg/kg dose of *B. balsamifera* leaf extract, ^d^ compared to 400 mg/kg dose of *B. balsamifera* leaf extract, ^e^ compared to 600 mg/kg dose of *B. balsamifera* leaf extract. Differences were considered statistically significant at *p* < 0.05.

**Table 5:** Effect of the ethanolic leaf extract of *B. balsamifera* on relative liver and spleen weights in *P. berghei* ANKA-infected mice following the Peters’ four-day suppressive test.

Group	Relative Liver Weight (g/g Body Weight)	Relative Spleen Weight (g/g Body Weight)
Uninfected control	0.067 ± 0.008	0.002 ± 0.001 ^b,d,e,f^
*P. berghei*-infected	0.074 ± 0.004	0.008 ± 0.001 ^a,c^
Chloroquine (25 mg/kg)	0.069 ± 0.001	0.004 ± 0.001 ^b^
*B. balsamifera* ethanolic leaf extract (200 mg/kg)	0.075 ± 0.006	0.007 ± 0.001 ^a^
*B. balsamifera* ethanolic leaf extract (400 mg/kg)	0.070 ± 0.072	0.006 ± 0.006 ^a^
*B. balsamifera* ethanolic leaf extract (600 mg/kg)	0.072 ± 0.003	0.006 ± 0.001 ^a^

Data are presented as the mean ± standard deviation (SD) (n = 5 mice per group). ^a^ Compared to uninfected, ^b^ compared to *Pb*A-infected, ^c^ compared to standard chloroquine, ^d^ compared to 200 mg/kg dose of *B. balsamifera* leaf extract, ^e^ compared to 400 mg/kg dose of *B. balsamifera* leaf extract, ^f^ compared to 600 mg/kg dose of *B. balsamifera* leaf extract. Differences were considered significant at *p* < 0.05.

**Table 6:** Effect of the ethanolic leaf extract of *B. balsamifera* on body weight changes in *P. berghei* ANKA-infected mice during the Peters’ four-day suppressive test.

Group	Mean Body Weight (g)		% Changes
	Day 0	Day 4	
Uninfected control	38.78 ± 1.12	39.92 ± 1.10	2.83 ± 1.64 ^b^
*P. berghei* ANKA-infected	39.46 ± 1.58	37.99 ± 1.71	−3.94 ± 3.48 ^a,f^
Chloroquine (25 mg/kg)	41.06 ± 3.30	41.51 ± 3.70	2.21 ± 1.49
*B. balsamifera* ethanolic leaf extract (200 mg/kg)	38.43 ± 1.41	38.68 ± 2.44	0.48 ± 2.74
*B. balsamifera* ethanolic leaf extract (400 mg/kg)	38.83 ± 2.70	39.17 ± 2.35	0.89 ± 2.12
*B. balsamifera* ethanolic leaf extract (600 mg/kg)	39.80 ± 2.39	40.92 ± 2.34	2.72 ± 2.51 ^b^

Data are presented as the mean ± standard deviation (SD) (n = 5 mice per group). Day 0 represents the day of infection, and Day 4 represents the end of the treatment period. Superscript letters indicate statistically significant differences (*p* < 0.05): ^a^, compared with the uninfected control group; ^b^, compared with the *P. berghei* ANKA-infected group; and ^f^, compared with the *B. balsamifera* ethanolic leaf extract (600 mg/kg) group.

**Table 7:** Effect of the ethanolic leaf extract of *B. balsamifera* on food consumption and water intake during the 14-day acute toxicity evaluation.

Group	Food Consumption (g)		Water Intake (mL)	
	Week 1	Week 2	Week 1	Week 2
Normal control	32.57 ± 1.49	32.28 ± 1.66	40.40 ± 6.74	40.00 ± 6.54
Vehicle control (7% Tween 80)	31.57 ± 0.72	32.57 ± 1.76	42.42 ± 5.12	38.14 ± 4.38
*B. balsamifera* ethanolic leaf extract (2000 mg/kg)	30.85 ± 1.55	30.85 ± 1.55	37.57 ± 6.52	36.42 ± 4.40

Data are presented as the mean ± standard deviation (SD) (n = 5 mice per group). Differences among groups were considered statistically significant at *p* < 0.05.

**Table 8:** Effect of the ethanolic leaf extract of *B. balsamifera* on body weight changes during the 14-day acute toxicity evaluation.

Group	Mean Body Weight (g)		
	Day 0	Day 14	% Change
Normal group	33.79 ± 0.92	42.10 ± 1.36	19.73 ± 1.99
Vehicle group (7% Tween 80)	33.77 ± 1.68	42.37 ± 3.57	20.30 ± 3.79
*B. balsamifera* ethanolic leaf extract (2000 mg/kg)	32.28 ± 0.59	38.78 ± 1.09	16.73 ± 3.00

Data are presented as the mean ± standard deviation (SD) (n = 5 mice per group). Day 0 represents the baseline measurement before extract administration, and Day 14 represents the end of the observation period. Differences among groups were considered statistically significant at *p* < 0.05.

**Table 9:** Effect of the ethanolic leaf extract of *B. balsamifera* on serum biochemical parameters associated with liver and kidney function during the 14-day acute toxicity evaluation.

Biochemical Parameters	Normal Group	Vehicle Group (7% Tween 80)	*B. balsamifera* Ethanolic Leaf Extract (2000 mg/kg)
**Live function parameters**			
AST (U/L)	111.00 ± 9.93 ^b,c^	78.00 ± 10.80 ^a^	82.60 ± 6.08 ^a^
ALP (U/L)	107.00 ± 23.23	116.00 ± 25.19	105.40 ± 18.88
ALT (U/L)	42.00 ± 12.56	26.80 ± 8.86	24.00 ± 6.16
**Kidney function parameters**			
BUN (mg/dL)	10.39 ± 6.58	10.64 ± 4.51	13.75 ± 2.92
Creatinine (mg/dL)	0.48 ± 0.26	0.62 ± 0.12	0.50 ± 0.13

Data are presented as the mean ± standard deviation (SD) (n = 5 mice per group). Superscript letters indicate statistically significant differences (*p* < 0.05): ^a^, compared with the normal control group; ^b^, compared with the vehicle control group; and ^c^, compared with the *B. balsamifera* ethanolic leaf extract (2000 mg/kg) group.

## Data Availability

The original contributions presented in this study are included in the article. Further inquiries can be directed to the corresponding author.
